# Optimal sample type and number vary in small shallow lakes when targeting non-native fish environmental DNA

**DOI:** 10.7717/peerj.15210

**Published:** 2023-05-02

**Authors:** Maïlys H.V. Picard, Anastasija Zaiko, Annabel M. Tidy, David J. Kelly, Georgia Thomson-Laing, Shaun P. Wilkinson, Xavier Pochon, Marcus J. Vandergoes, Ian Hawes, Susanna A. Wood

**Affiliations:** 1 School of Biological Sciences, Department of Biological Sciences, University of Waikato, Hamilton, New Zealand; 2 Coastal and Freshwater, Cawthron Institute, Nelson, New Zealand; 3 Institute of Marine Science, University of Auckland, Warkworth, New Zealand; 4 University of Birmingham, Birmingham, United Kingdom; 5 Wilderlab NZ Ltd, Wellington, New Zealand; 6 GNS Science, Lower Hutt, New Zealand

**Keywords:** Non-native species, Fish, Environmental DNA, Sedimentary DNA, Occupancy modelling, Sampling design, *Perca fluviatilis*, *Scardinius erythrophthalmus*, Lake

## Abstract

Non-native fish have been shown to have deleterious impacts on freshwater ecosystems in New Zealand. Early detection is critical for their effective management. Traditional capture-based techniques may not detect newly introduced fish, especially if they are present in low abundance. Molecular techniques that target environmental DNA (eDNA) have been shown, in many instances, to be more sensitive, cost-effective and require lower sampling effort. However, appropriate sampling strategies are needed to ensure robust and interpretable data are obtained. In this study we used droplet digital PCR assays to investigate the presence of two non-native fish in New Zealand, the European perch (*Perca fluviatilis*) and rudd (*Scardinius erythrophthalmus*) in three small lakes. Samples were collected from water and surface sediment at near-shore and mid-lake sites. Probabilistic modelling was used to assess the occupancy of fish eDNA and develop guidance on sampling strategies. Based on the detection probability measures from the present study, at least six sites and five replicates per site are needed to reliably detect fish eDNA in sediment samples, and twelve sites with eight replicates per site for water samples. The results highlight the potential of developing monitoring and surveillance programs adapted to lakes, that include the use of assays targeting eDNA. This study focused on small shallow lakes, and it is likely that these recommendations may vary in larger, deeper, and more geomorphologically complex lakes, and this requires further research.

## Introduction

The introduction of non-native fish species into aquatic ecosystems can pose a major threat to local biodiversity. Once established they can disrupt food webs through top-down trophic cascades (Dextrase & Mandrak, 2006; Bellard, Cassey & Blackburn, 2016) and reduce water quality with adverse consequences for ecosystem services, human health, and the economy (Keller et al., 2009; Pejchar & Mooney, 2009; Strayer, 2010; Ricciardi et al., 2013). A well-known example is the global spread of carp (*Cyprinus carpio*). In many lakes, their introduction has caused degradation in water quality with lakes flipping from clear water, macrophyte-dominated to turbid, phytoplankton-dominated, which has resulted in the loss of native biodiversity (Williams, Moss & Eaton, 2002; Parkos III, Santucci Jr & Wahl, 2003; Koehn, 2004; Miller & Crowl, 2006; Kloskowski, 2011). Similarly, multiple non-native species such as the brown bullhead (*Ameiurus nebulosus*) and pumpkinseed (*Lepomis gibbosus*) have been introduced in North America, degrading waterways and changing food webs (Dextrase & Mandrak, 2006). To mitigate the impact of non-native fish, limiting their spread is paramount. The likelihood of successful control or eradication is enhanced if non-native species are detected soon after incursion and if their spread can be effectively monitored to measure the success of management response (Hulme, 2006; Britton, Gozlan & Copp, 2011; Rytwinski et al., 2018).

Newly introduced species are usually not very abundant, and early detection is a critical first step that may assist in effective management (Mehta et al., 2007; Roux & Wieczorek, 2009; Britton, Davies & Harrod, 2010). However, traditional approaches to detect and monitor fish (*e.g.*, nets, electrical fishing, spotlighting) are time-consuming, costly, and can underestimate their spread or completely overlook their presence (Sigsgaard et al., 2015; Thomsen & Willerslev, 2015). Recent molecular approaches which target environmental DNA (eDNA) are promising tools for effective and non-intrusive surveying of fish (Darling & Mahon, 2011). Environmental DNA refers to the genetic material from whole cells or released by organisms in the form of tissue slough, shed or lysed cells, gametes, excretion and saliva, among other secretions (Taberlet et al., 2012; Taberlet et al., 2018; Pawlowski, Apothéloz-Perret-Gentil & Altermatt, 2020). Approaches that target eDNA are often more sensitive than traditional methods (Jerde et al., 2011; Ardura et al., 2015; Gantz et al., 2018), are not directly reliant on taxonomic expertise, and relatively simple, standardized sampling and analytical protocols can be developed. Quantitative PCR techniques also allow the precise quantification of target gene copies in environmental samples, and in some instances eDNA concentrations have been shown to be indicative of species biomass (Takahara et al., 2012; Doi et al., 2015; Eichmiller, Miller & Sorensen, 2016; Lacoursière-Roussel et al., 2016; Capo et al., 2019; Rourke et al., 2022).

To date, most studies targeting eDNA to detect or track aquatic species have collected and analyzed water samples (Rees et al., 2014), with a key rationale being that DNA degrades relatively rapidly (within days to weeks) therefore detection in these samples likely indicates recent presence (Buxton, Groombridge & Griffiths, 2017). However, given the mobility of fish and the labile nature of DNA, studies have shown that fish eDNA can be patchy in water samples (Eichmiller, Bajer & Sorensen, 2014; Lawson Handley et al., 2019). Targeting surface sediment may allow for a more time-integrated approach, providing information on the presence of fish over the last few months to years. Some studies to date indicate higher levels and slower decay rates in sediment compared to water (Eichmiller, Bajer & Sorensen, 2014; Turner, Uy & Everhart, 2015; Sakata et al., 2020), while others have shown higher detection in water compared to sediment samples (Buxton, Groombridge & Griffiths, 2018; Valdez-Moreno et al., 2019). Further research is required to compare the detection of fish eDNA between water and sediment samples and to gain further insights into how this varies among species to support and optimize the use of eDNA approaches for non-native fish surveillance.

While many studies have been published on the application of eDNA monitoring for fish in rivers (*e.g.*, Pont et al., 2018; Cantera et al., 2019; Rourke et al., 2022), there is less data on the spatial variation of eDNA within lakes or on how to develop appropriate sampling designs. Depending on the season, fish may occupy different habitats (*e.g.*, pelagic or littoral), be more or less active and the water column may be stratified or fully mixed, all of which affects eDNA dispersal (Klobucar, Rodgers & Budy, 2017; Lawson Handley et al., 2019; Littlefair et al., 2021). Occupancy modelling has now been applied to data generated from eDNA surveys, to assist in understanding such spatial and temporal detectability variations (Schmelzle & Kinziger, 2016; Smith & Goldberg, 2020; McClenaghan, Compson & Hajibabaei, 2020; Steiner et al., 2023). Occupancy modelling estimates the distribution of a target (such as a species or its eDNA) in a given environment and the probability that it will be detected, while accounting for imperfect detection.

New Zealand is an island nation in the Southwest Pacific that has been isolated from other landmasses for about 85 million years (Daugherty, Gibbs & Hitchmough, 1993). Isolation allowed unique flora and fauna to evolve, which have been heavily impacted by the introduction of non-native species over the last approximately 700 years (Towns, Simberloff & Atkinson, 1997). New Zealand’s freshwater systems have been subjected to multiple introductions over this period, with a range of documented consequences including decreases in water quality and predation on native fish (Rowe, 2007; McIntosh et al., 2010). Two fish species of considerable concern are the European perch (*Perca fluviatilis*, hereafter perch) and rudd (*Scardinius erythrophthalmus*). Perch are carnivorous coarse fish which were introduced for angling in the 1870s. This pelagic species is voraciously zooplanktivorous during its juvenile stage and becomes piscivorous as an adult. It is now well-established in many lowland lakes with causative links to declines in native fish and zooplankton communities (Romare, Bergman & Hansson, 1999; Rowe, 2007) and increases in cyanobacterial blooms (Smith & Lester, 2006; Smith & Lester, 2007). Rudd is a benthopelagic coarse fish which was introduced in 1967 (Department of Conservation, 2006). In New Zealand, the adults prefer to feed on native macrophytes, therefore their grazing impact has been linked to macrophyte collapse, with concomitant declines in water quality (Hicks, 2001; Lake et al., 2002). The feeding strategies of both rudd and perch differ from those of native fish, making New Zealand lake ecosystems particularly vulnerable to their introduction. It is now forbidden to sell or intentionally breed and propagate either species (Biosecurity Act 1993). However, perch is legally classified as a sport fish (Freshwater Fisheries Regulations 1983), while rudd is a noxious fish in all parts of the country except for the Auckland/Waikato regions where it is considered a sport fish. Although the spread of perch and rudd between lakes is now limited, there continues to be intentional and accidental secondary introductions across the country (Mitchell, 2020).

The overall goal of this study was to optimize the use of assays targeting eDNA for the detection of non-native fish in small, shallow lakes. The aims of this study were to determine; (1) which sample type, water or surface sediment, and which location, near-shore or mid-lake, would be best suited to detect perch and rudd in small shallow lakes, and (2) how many sites and replicates would be needed to reliably detect fish eDNA. Water and sediment samples were collected from fourteen sites in three lakes. Species-specific droplet digital PCR assays were used to detect eDNA and the results analyzed using occupancy modelling. Sampling was designed to test three hypotheses, being (1) eDNA will be homogeneously distributed across lakes for both species but due to their life history (benthic rudd *versus* pelagic perch), (2) rudd eDNA will be better detected in sediment samples while (3) perch eDNA will be better detected by water samples.

## Materials & Methods

### Sampling sites

**Figure 1:**
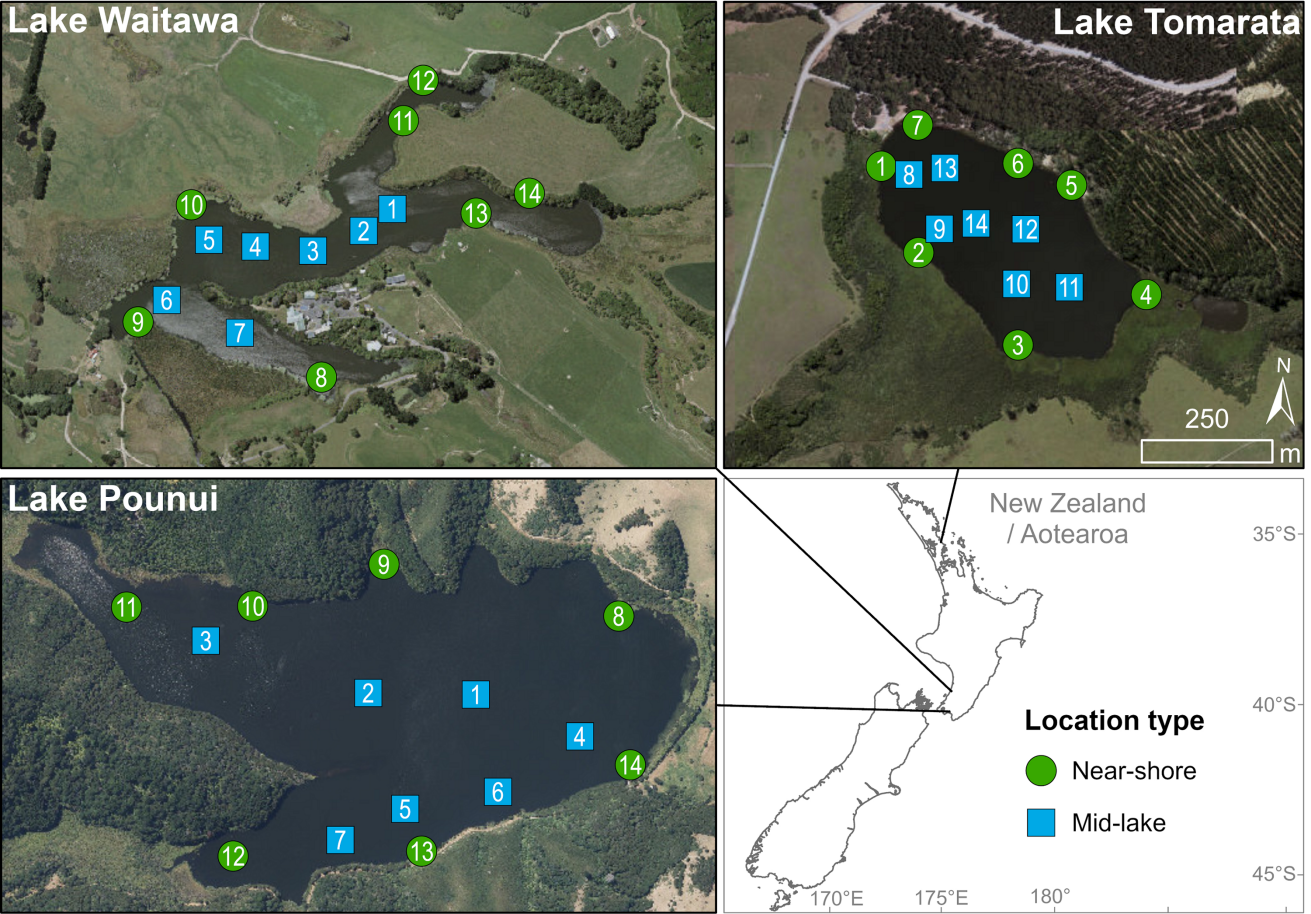
Sampling sites and location (near-shore *vs* mid-lake) in each lake. Created with ArcGIS 2.9 Desktop by Lisa Floerl (Cawthron Institute).

Three small, lowland lakes in the North Island of New Zealand were sampled: Pounui, Waitawa and Tomarata (Fig. 1, Table S1). Fourteen sites were sampled in each lake using a small motorboat or canoe, seven near the shore (generally <3 m from the shoreline) and seven near the middle of the lake (>7 m from the lake edge, Fig. 1, Table S1). Field work was undertaken in spring, with cyanobacterial blooms observed in Lakes Pounui and Waitawa during sampling. Previous catch data indicate that Lake Waitawa has two to three times more perch than rudd, and Lake Waitawa has about three times more perch than Lake Pounui (Drake, Kelly & Schallenberg, 2011; Alton Perrie pers. comm., 2019). Further lake characteristics and information on the presence of native and non-native fish are provided in Table 1. All samples were collected under the specifications of Special Permit 651 from the New Zealand government agency Ministry for Primary Industries.

**Table 1:** Lake characteristics and fish community composition. Descriptions of the land cover in the catchments were derived from the Land Cover Database Version 5 (Landcare Research New Zealand Ltd). Sources for fish catch data are: 1. Drake, Kelly & Schallenberg (2011). 2. Greater Wellington Regional Council monitoring (Alton Perrie pers. comm., 2019).

	**Pounui**	**Tomarata**	**Waitawa**
Sampling date	4 December 2018	18 November 2018	13 October 2019
Location (Lat/Long)	41° 20′41.3″S 175° 06′47.7″E	36° 11′36.0″S 174° 39′00.3″E	40° 43′28.3″S 175° 10′21.6″E
Lake area (km^2^)	0.46	0.144	0.158
Max depth (m)	9.6	5.6	5
Main catchment vegetation	Native vegetation (83%)	High producing grasslands (>50%)	High producing grasslands (>90%)
Non-native fish	European perch (*Perca fluviatilis*), brown trout (*Salmo trutta*)	Goldfish (*Carassius auratus*), Koi carp –may be eradicated (*Cyprinus rubrofiscus*), rudd (*Scardinius erythrophthalmus*), tench (*Tinca tinca*), rainbow trout –stocked (*Oncorhynchus mykiss)*	European perch (*P. fluviatilis*), goldfish (*C. auratus*), Koi carp (*C. rubrofiscus*), rudd (*S. erythrophthalmus*), tench (*T. tinca*)
Native fish	Longfin eel (*Anguilla dieffenbachii*)^1,2^, shortfin eel (*Anguilla australis*)^1,2^, common bully (*Gobiomorphus cotidianus*)^1,2^, ı¯nanga (*Galaxias maculatus*) ^1^	Longfin eel (*A. dieffenbachii*) ^1^, shortfin eel (*A. australis*) ^1^, common bully (*G. cotidianus*) ^1^, ı¯nanga (*G. maculatus*) ^1^	Longfin eel (*A. dieffenbachii*)^1,2^, shortfin eel (*A. australis*)^1,2^, common bully (*G. cotidianus*)^1,2^

### Sediment geochemistry

A single surface sediment sample was collected using a Ponar grab from the deepest part of each lake. The top two cm of the grab were collected using spatulas and placed in 500 mL containers. Sediment was homogenized, stored chilled (4 °C) and shipped to the laboratory within 48 hrs for nutrient and elemental characterization. Once in the laboratory, sediment was homogenized again, centrifuged (3,000×g, 40 min, 4 °C), and the pore water decanted. Leftover sediment was dried and passed through a sieve (two mm) for metal analysis using acid digestion followed by Inductively Coupled Plasma-Mass Spectrometry (ICP-MS) analysis based on the US Environmental Protection Agency (EPA) method 200.8. The metals analyzed were aluminum (Al), calcium (Ca), cadmium (Cd), copper (Cu), iron (Fe), manganese (Mn), phosphorus (P), lead (Pb), zinc (Zn) and sulfur (S). Reporting limits (mg kg-1) were: 12.5, 0.125, 2.5, 12.5, 0.05, 0.075, 0.05, 0.005, 10 and 250, respectively. Total Organic Carbon (TOC) and Total Nitrogen (TN) were analyzed using catalytic combustion at 900 °C (O2) and separation using a thermal conductivity detector (reporting limit for both g/100g). Organic matter was measured using over drying, ashing (550 °C), and gravimetric determination. Grain size distributions were determined using a laser diffraction particle size analyzer at the University of Waikato (Hamilton, New Zealand).

### Rudd assay development

Two primers and a probe were designed to target rudd (*Scardinius erythrophthalmus*) without cross-amplifying native and other exotic fish that are present in New Zealand. Rudd sequences (16S mitochondrial DNA) were aligned with 87 fish sequences using the aphid R package (Wilkinson, 2019), and primers and probe designed with the Geneious software (Geneious, 2019) (Fig. S1). The resulting amplicon was 101 bp (Table 2).

**Table 2:** Primer pairs used in this study. The amplicon length in base pairs (bp) are indicated for each marker gene. The rudd assay was tested on other fish DNA extracts and samples of known composition to check the specificity of the assay in-vivo (Tables S2,  S3).

Target	Marker	Primer name	Sequence	Source
*Perca fluviatilis* 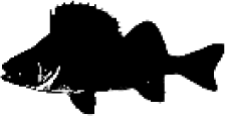	12S rRNA gene (92 bp)	P.flu_12S_F P.flu_12S_R P.flu_12S_P	Forward: 5′-GGGATTAGATACCCCACTATGCCT-3′ Reverse: 5′-GGTTTCAAGCTGATGCTCGTAGTT-3′ Probe: 5′-(FAM)-CCATAAACATTGGTAGCACACT-(MGB)-3′	Furlan & Gleeson (2016)
*Scardiniuserythrophthalmus* 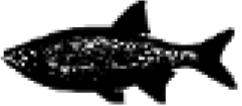	16S rRNA gene (188 bp)	S.eu_16S_F S.eu_16S_R S.eu_16S_F1	Forward: 5′-CACGTTAAACGACTCTGTAG-3′ Reverse: 5′-GTTCGTTGATCGGCTTTATCAGT- 3′ Reverse: 5′-GTTCGTTGATCGGCTTTATCAGT- 3′	This study

In-vivo, this primer set successfully amplified rudd DNA and did not cross-amplify the DNA of eleven other fish species (Tables S2,  S3). Specifically, this rudd assay did not amplify genomic extracts of brown bullhead catfish, goldfish, koi carp, Gambusia, European perch, and tench (Table S2). Environmental DNA samples were also tested with this rudd assay (qPCR), and then sequenced with an iSeq instrument (metabarcoding). This comparison revealed that the present rudd assay was able to detect rudd eDNA when it was present in the sample, and yielded no detection when rudd DNA was absent in the sample. Using this method, this assay did not cross-amplify the eDNA of brown bullhead catfish, short-finned eel, goldfish, gambusia, skipjack tuna, rainbow trout, smelt, brown trout, and tench, which were present in the samples when rudd detection was negative (Tables S2, S3).

### Environmental DNA sample collection

Two sample types were collected at each site: surface water (hereafter referred to as water samples) and lakebed surface sediment (hereafter referred to as sediment samples). Samples were taken in duplicate at each site, *i.e.,* a total of 28 water and 28 sediment samples from each lake, for a total of fifty-six samples per lake. Water samples were collected in 1L plastic bottles from the lake surface. They were kept refrigerated during transport and filtered within 24 h (GF/C filters, pore size ∼1.6 µm, 47 mm dia., Whatman, UK). Two negative controls (tap water) were also included at the beginning and end of filtering for each lake. The filters were halved with sterilized scissors, the two half filters placed in separate Eppendorf tubes and immediately frozen (−20 °C). Water samples were collected prior to sediment sampling for every site to avoid contaminating the water samples with sediment. Due to the presence of heavy cyanobacterial blooms in Lake Waitawa, water samples were pre-filtered through a 50 µm mesh to remove particles likely to clog the GF/C filters. Sediment samples were collected using a Ponar grab which was dropped two times. Undisturbed surface sediment (∼3 g) layer (max. 0.5 cm depth) was sampled using a sterilized spatula and placed in sterile five mL tubes. Samples were chilled during transport and frozen (−20 °C) within 2 h. Sampling equipment was soaked in 2% bleach solution (>2 min) and rinsed three times in lake water between sampling sites. Filtering equipment was also soaked in 2% bleach solution (>2 min) and rinsed three times with tap water between filtering different samples.

### DNA extraction

Sediment samples up to 3 g were extracted using a combination of an alkaline lysis method with ethanol precipitation followed by the DNeasy PowerSoilTM kit (Qiagen, USA), as detailed in Thomson-Laing et al. (2022). For the lysis step, sodium hydroxide (6 mL, 0.33M) and Tris-EDTA (3 mL, pH 8) were added to the sediment samples in sterile 15 mL tubes. The tubes were thoroughly mixed by benchtop vortex (1 min), and incubated (65 °C, 50 min). The samples were cooled to room temperature and centrifuged (3,214 g, 1 h). Part of the supernatant (7.5 mL) was transferred to a sterile 50 mL tube and the lysis step neutralized with the same volume of Tris HCl (7.5 mL, 1 M, pH 6.7). For the precipitation step, sodium acetate (1.5 mL, 3M, pH 5.2) and ethanol (30 mL, molecular grade 100%) were added in the same tube and samples were stored at −20 °C (>12 h). Finally, samples were centrifuged (3,200×g, 1 h) and the supernatant discarded. These extraction steps were undertaken in batches of 20 including a blank control every second batch.

Following ethanol precipitation, the entire sediment pellet (up to 0.5 g) was transferred to the bead beating tube of a DNeasy PowerSoilTM kit (Qiagen, USA) using a sterilized spatula. Similarly, one half of the filters from the water samples was cut into smaller pieces and transferred to bead beating tubes. DNA was extracted from the filters and sediment pellet using the DNeasy PowerSoilTM kit (Qiagen, USA) and the automated QIAcube instrument (Qiagen, USA) following the manufacturer’s protocol. Samples were extracted in batches of 10–12 samples, with extraction controls included every two batches (same controls as the pre-extraction method).

### Single-species quantification with droplet digital PCR

Each following step was conducted in a separate sterile laboratory, with sequential workflow to ensure no cross-contamination. Rooms dedicated to PCR set-up and template addition were equipped with ultra-violet sterilization which was switched on for a minimum of 15 min before and after each use. The ddPCR set-up and template addition were undertaken in laminar flow cabinets with HEPA filtration. Aerosols barrier tips (epT.I.P.S.; Eppendorf, Hamburg, Germany) were used throughout.

Droplet digital PCR was used to quantify target mitochondrial genes from perch (12S rDNA) and rudd (16S rDNA; Table 2). Following the recommendations of BioRad when applying ddPCR probe assays on environmental samples, two restriction enzymes were used to help cleave non-target DNA: HindIII-HF and HaeII (New England Biolabs, Ipswich, MA, United States). All primers and probes were run in duplex ddPCR analyses, using a BioRad QX200 system. Each ddPCR reaction included 1 µL (10 µM) of each primer and probe, 10 µL of 1 × ddPCR Supermix for probes with no dUTP (Bio-Rad, Hercules, CA, United States), 3 units of each restriction enzyme, and 6 µL of template DNA for a total reaction volume of 22.45 µL.

The BioRad QX200 droplet generator mixed 20 µL of the reaction mixture with 70 µL of BioRad probe droplet oil. The final volume of 40 µL contained c. 20,000 nanodroplets and was transferred to a semi-skirted ddPCR 96-well plate for amplification using the following cycling protocol: hold at 95 °C for 10 min, 39 cycles of 94 °C for 30 s, 57 °C for 1 min, and a final enzyme deactivation step at 98 °C for 10 min. Finally, the plate was transferred to the BioRad QX200 droplet reader to count how many PCR-positive and PCR-negative nanodroplets were present in each sample. One negative control (all reagents and RNA/DNA-free water) and one positive control (DNA extracted from perch and rudd tissue samples) were included in each ddPCR run.

To take into account the possibility of PCR inhibition, any sample with less than 10,000 total droplets was re-run. The minimum number of droplets in any sample in this study was 18,244. Furthermore, following the methods from Dingle et al. (2013), a broad amplitude threshold was defined to account for inhibited positive droplets. In short, the threshold for positive droplets was set just above the cloud of negative droplets to account for variations in the amplitude of positive droplets. Last, if only one positive droplet was detected, the sample was re-run twice to help avoid false-positive detections. The sample was considered positive if two out of the three final technical replicates were positive, and the final concentration was calculated as the mean of the two positive technical replicates. Droplets counts were normalized with a Poisson distribution by the QuantaSoft Analysis software (BioRad), and are reported as the concentration of gene copies (copies/µL) per ddPCR reaction.

Thresholds to differentiate positive *vs* negative droplets were determined for both assays by diluting the positive controls and running negative controls alongside. Amplitude thresholds were set at 2,734 for perch and 1,735 for rudd, as per methods from Capo et al. (2019). The limit of quantification (LoQ) of the duplex assay was calculated as per methods used in Brys et al. (2021) and Picard et al. (2022). The combined perch and rudd DNA extracts used as positive controls were measured on a nanofluorometer and diluted in 6 steps (1 ng/µL down to 10^−5^ ng/µL). These dilutions were measured on the ddPCR system using the duplex perch/rudd assay with three replicates per dilution step. The calculated concentrations (ng/µL) were then plotted against the measured concentrations (copies/µL) using a LOESS smoothing function, and the LoQ was determined as the threshold were the measured trend started to differ from the expected trend (Fig. S2).

### Data analysis

Data analysis and plots were performed with the R (R Core Team, 2021) and RStudio software (RStudio Team, 2022), using the Tidyverse and its associated packages (Wickham et al., 2019). Raw ddPCR concentrations as well as weight- or volume-normalized ddPCR concentrations were compared across lakes, species, sample types, and locations using boxplots. To enable the comparison of eDNA levels across sample type, one mL of water was assumed to equal 1 g so that both samples type could be plotted as gene copy numbers per gram (Sakata et al., 2020). The data were neither normally distributed nor homoscedastic and included null values, therefore it was transformed (exp for raw concentrations and log10 +1 for normalized concentrations) for visualization and statistical testing. Overall differences across locations and across sample type depending on the fish species were tested using the non-parametric Kruskal-Wallis test, as well as differences across locations and sample type for a given lake.

Occupancy modelling was used to estimate the probability of target eDNA being present at a given sampling site and detection probability for a given lake under a range of sampling strategies. Briefly, the models were used to test which combination of sampling method and location was best to detect fish eDNA in each lake. The environmental DNA levels were transformed into presence-absence for all biological replicates to run the occupancy models and sampling design simulations to optimize for the best detection probabilities for each lake and fish species. Since perch eDNA was only detected in one sample in Lake Tomarata, perch data from this lake were excluded from further analysis. Occupancy models were undertaken for perch eDNA in Lakes Pounui and Waitawa and rudd eDNA in Lakes Tomarata and Waitawa. Occupancy modelling was performed using the PRESENCE v12.31 software (MacKenzie et al., 2002; Nichols et al., 2008) to estimate the eDNA occupancy (Ψ) and the detection probability (p) for each target fish per lake (see details below). A single-season multi-method model variant was applied to compare sediment and water samples.

The following parameters were estimated in each model:

*ψ*naïve = naïve estimate of occupancy probability or proportion of area occupied (PAO), calculated as the number of sites where fish eDNA was detected over all sites surveyed;

Ψ = large-scale occupancy, *i.e.,* probability of a sample unit being occupied by a target eDNA;

*θ* = small-scale occupancy or model-estimated occupancy, *i.e.,* the probability that the target eDNA is present in the direct vicinity of the sampler and available for collection at a sampling time, given that sample unit is occupied;

p = probability of target eDNA detection at a sampling time by method m, given that sample unit is occupied, and target eDNA is present at immediate sampling location.

Sampling sites (*n* = 14) were treated as sampling units and biological replicates (*n* = 2 for sediment and water, total *n* = 4) as repeated surveys. In the predefined models, detection probabilities were assessed for each sampling method (*i.e.,* water *vs.* sediment) and location within the lake (*i.e.,* mid-lake *vs.* near-shore, *n* = 7 each). All model combinations (hereafter models variants) and their rankings for each species and lake can be found in Tables S5–S8.

Model variants were ranked by the PRESENCE software according to their Akaike Information Criterion (AIC) values, and the lowest AIC was used to select the best model for each species per lake. Several parameters were extracted from the model summary (*ψ*naïve, Ψ, *θ*, p) to understand the distribution and detection probability of fish eDNA in each lake. Code from Guillera-Arroita, Ridout & Morgan (2010) was then used to run simulations of detection histories in RStudio. Briefly, the simulations allowed us to specify several parameters (Ψ, p, number of sites sampled, number of replicates) to test whether the number of sites and replicates were adequate to detect the target given a specific occupancy (Ψ) and detection probability (p). We used these simulations to estimate how many sites and replicates were needed in a worst-case monitoring scenario (*i.e.,* using the lowest probability detections) to detect fish eDNA using ddPCR in lakes of similar size. Simulations were run 10,000 times as described in Guillera-Arroita, Ridout & Morgan (2010), and since all occupancy models showed constant occupancy, the Ψ parameter was set at 1 (100%). Avoiding false negative detection (*i.e.,* complete lack of false negatives across all sites and replicates within the lake) was selected as the most important criterion (estimated empty histories = 0%), and the best simulation (per sample type) identified minimum meaningful sampling effort while reducing the potential bias, with less than 5% of standard error rates on the estimates and on false negative detections.

## Results

### Sediment geochemistry

The sediment in the mid-lake zone of Lake Waitawa had a fine gelatinous consistency which was not observed in the other two lakes. Waitawa sediments were very low density (21 kg.m-3 dry weight) compared to medium density for Lakes Tomarata and Pounui (respectively 205 kg.m-3 and 128 kg.m-3). Grain size was skewed away from fine particulates with high surface area to mass. Only 28% of the Waitawa sediment had a grain size ≤ 63 µm compared to 58% in Tomarata (no results for Pounui). A relatively high organic to ash content was also measured for Waitawa and overall low concentrations of iron (Table S4).

### Overall detections

**Figure 2:**
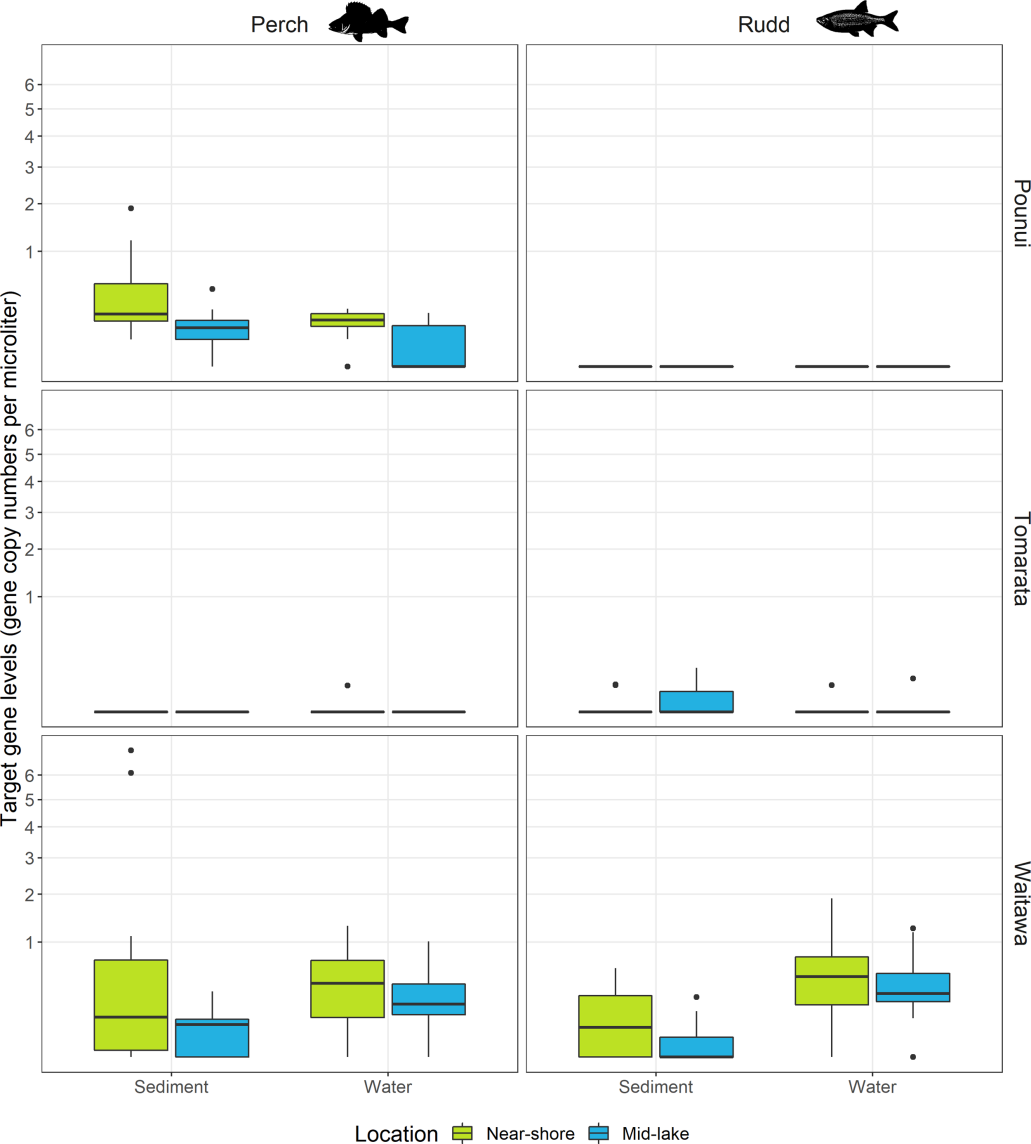
Target gene levels for each fish species per lake (12S rDNA for perch and 16S rDNA for rudd), separated by sampling method (sediment and water) and sampling location (near-shore and mid-lake). Gene levels are presented as raw values (gene copies/µL of ddPCR reaction), extracted from c. 3 g sediment and 500 mL of water. Gene levels are plotted on a square-root scale.

**Figure 3:**
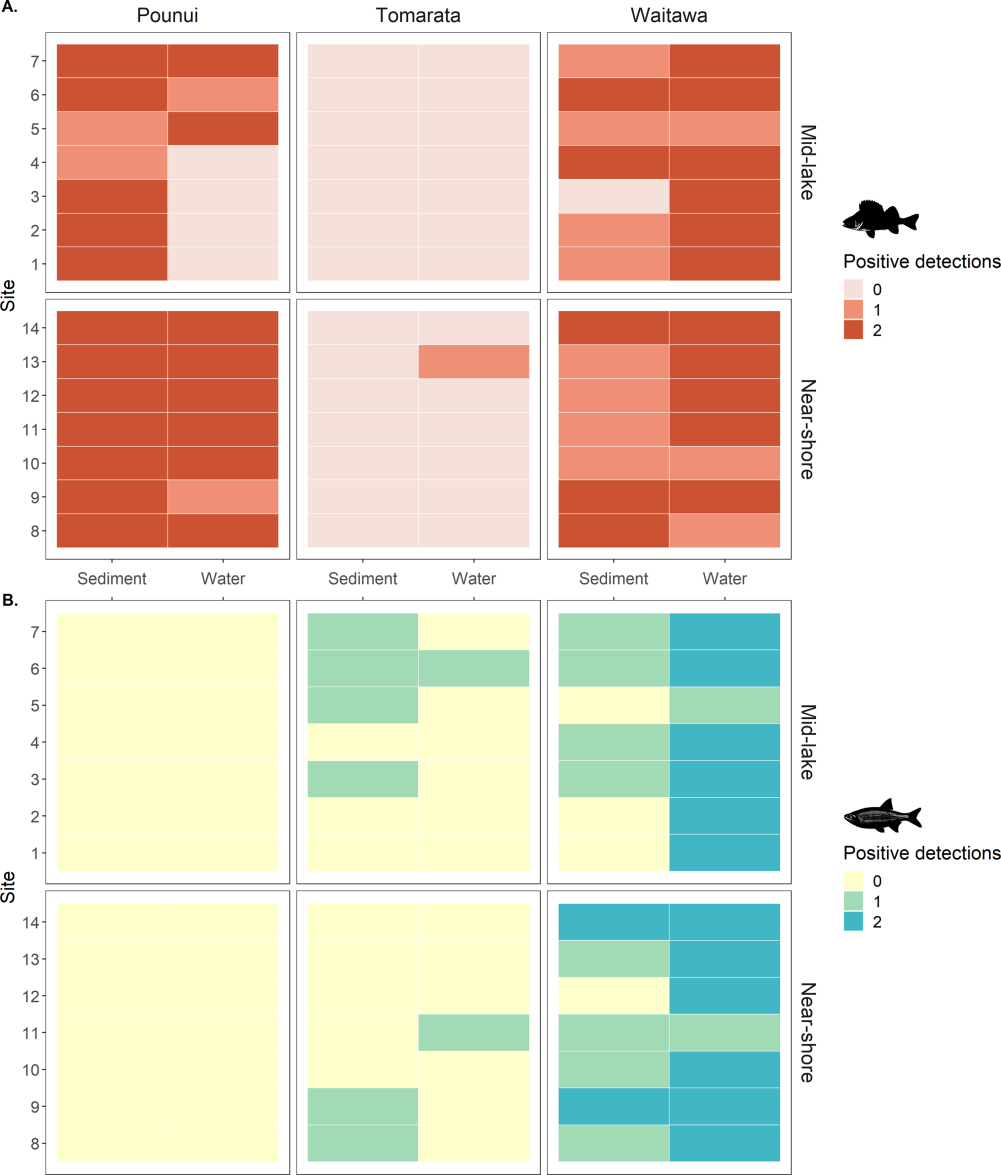
(A) Perch (*Perca fluviatilis*) and (B) Rudd (*Scardinius erythrophthalmus*) eDNA detection in each biological replicate across lakes, sites, location, and sample type. The genes targeted were 12S rDNA for perch and 16S rDNA for rudd.

The eDNA of the target species were not detected in any of the negative control samples. Perch eDNA was detected in Lakes Pounui, Tomarata, Waitawa. Rudd eDNA was detected in Lakes Tomarata and Waitawa. Fish eDNA was rarely detected at all sites for a given sample type except in sediment samples (perch) in Lake Pounui and water samples for Lake Waitawa (perch and rudd, Figs. 2 and 3). Fish eDNA levels varied greatly depending on the lake and sample type, from 0.05 to 1.9 gene copies/µL of ddPCR reaction in water samples and from 0.05 to 7.1 gene copies/µL of ddPCR reaction in surface sediment (Fig. 2). The Limit of Quantification (LoQ) was calculated at 13 copies/uL for perch and 5 copies/uL for rudd (Fig. S2), and the highest eDNA levels were in Lake Waitawa for both sample type and both species (Fig. 2). Environmental DNA levels were normalized to gene copy numbers per liter or per gram in Fig. S3.

### Detection comparison for Perch and Rudd

Perch eDNA was detected in 52% of all biological replicates (88 out of 168 samples) across all lakes and at 29 out of 42 sites (69%). This included all sites in Lakes Pounui and Waitawa, and one site in Lake Tomarata (although perch is not known to occur in this lake). Perch eDNA levels were significantly higher at near-shore sites *versus* mid-lake sites in Lake Pounui (Kruskal-Wallis test H 15.296, *df* = 1, *p* < 0.001, Fig. 2). Very high perch eDNA levels were detected in the sediment samples of Lake Waitawa (sites 9 and 12, 7 and 6 gene copies/uL, respectively) compared to other samples which were overall lower than 1 gene copy/uL (Fig. 2). Once these outliers were removed, eDNA levels were significantly higher in the water samples of Lake Waitawa compared to its sediment samples (Kruskal-Wallis test H 10.067, *df* = 1, *p*-value = 0.002).

In general, detection patterns for perch eDNA, as presence-absence, were similar among sediment and water samples with 79% similarity among sample types (no detection at 13 sites, detection in 23 sites by both, four only in sediment, two only in water). However, the trends were different within each lake (Fig. 3). In Lake Pounui, sediment samples yielded more detections than water (93% detection in sediment *vs.* 64% detection in water). Conversely, the opposite pattern was observed in Lake Waitawa (64% sediment, 89% water). Spatial patchiness, as evidenced by the number of sites where perch was not detected, varied by lake: eDNA was patchy in water for Lake Pounui and found at all sites by sediment, while the opposite pattern was observed for Lake Waitawa.

Rudd eDNA was detected in 27% of all biological replicates (46 out of 168 samples) and 21 of 42 sites (50%) across all lakes. The type of sample (sediment or water) yielding the highest detection varied between lakes, and there was only 52% similarity overall in detection among sample types (11 sites by both, five sediment only, five water only, Fig. 3). Detection was highest in sediment in Lake Tomarata (21% sediment and 7% water) while water yielded better detection in Lake Waitawa (43% sediment and 93% water). Rudd eDNA was patchier in sediment samples for Lake Waitawa with the opposite pattern observed at Lake Tomarata. Like for perch, rudd eDNA levels were significantly higher in water samples compared to sediment for Lake Waitawa (Kruskall-Wallis *H* = 20.699, *df* = 1, *p*-value <0.001) while there was no significant difference in Lake Tomarata.

### Occupancy modelling

Sixteen model variants were run for each lake and species (Tables S5–S8), accounting for different combinations of sample type and location effect on target eDNA occupancy and probability of detection. The models indicated that, across all samples, perch and rudd eDNA was detected across all sites (*ψ*naïve = 100%) when the species was present in the lake, while rudd eDNA was only detected at 50% of the sites in Lake Tomarata (Table 3). All best-ranking models indicated constant distribution of eDNA across the lakes (both small- and large-scale occupancies), irrespective of the sampling location (near-shore or mid lake).

**Table 3:** Parameters derived from the best occupancy model for each fish’s eDNA within each lake. The best model was selected by its Akaike Information Criterion, and the PRESENCE software also indicated the likelihood that it explained the data compared to the other models (see Tables S5,  S8). The detection probabilities for significant combinations of sample type and location identified by the model are shown, best combinations are in bold.

	**Pounui**	**Tomarata**	**Waitawa**	
Target	Perch	Rudd	Perch	Rudd
Likelihood compared to second-best model	96%	19%	61%	7%
Proportion of sites occupied (*ψ*_naïve_)	100%	50%	100%	100%
Large-scale occupancy (Ψ)	*Constant distribution* (1)	*Constant distribution* (1)	*Constant distribution* (1)	*Constant distribution* (1)
Small-scale occupancy (*θ*)	*Constant distribution* (1)	*Constant distribution* (1)	*Constant distribution* (1)	*Constant distribution* (1)
Detection probability	*Method-dependent (sediment) and location-dependent (near-shore)* Mid-lake sediment: *p* = 0.86 ± 0.09 **Near-shore sediment**: *p* = 0.99 ± 0.008 Mid-lake water: *p* = 0.35 ± 0.13 Near-shore water: *p* = 0.93 ± 0.06	*Method-dependent* **Sediment**: *p* = 0.21 ± 0.08 Water: *p* = 0.07 ± 0.05	*Method-* *dependent* Sediment: *p* = 0.64 ± 0.09 **Water**: *p* = 0.89 ± 0.06	*Method-* *dependent* Sediment: *p* = 0.43 ± 0.09 **Water**: *p* = 0.93 ± 0.05

Detection probabilities varied depending on the sampling method and location. They were method-dependent for rudd eDNA in Lake Tomarata (sediment better than water in best and second-best models) and for perch and rudd eDNA in Lake Waitawa (water better than sediment). Perch eDNA in Lake Pounui was most likely to be detected in near-shore sediment samples (Table 3). The second-best model for rudd in Lake Waitawa ranked only 7% behind the first one and indicated that detection probabilities could be method and location-dependent, with near-shore water samples yielding the best detection. The probability of detecting fish eDNA was the highest (across the whole dataset) in near-shore sediment samples in Pounui (*p* = 0.99 ± 0.008), and lowest in water samples in Lake Tomarata (*p* = 0.07 ± 0.05).

**Figure 4:**
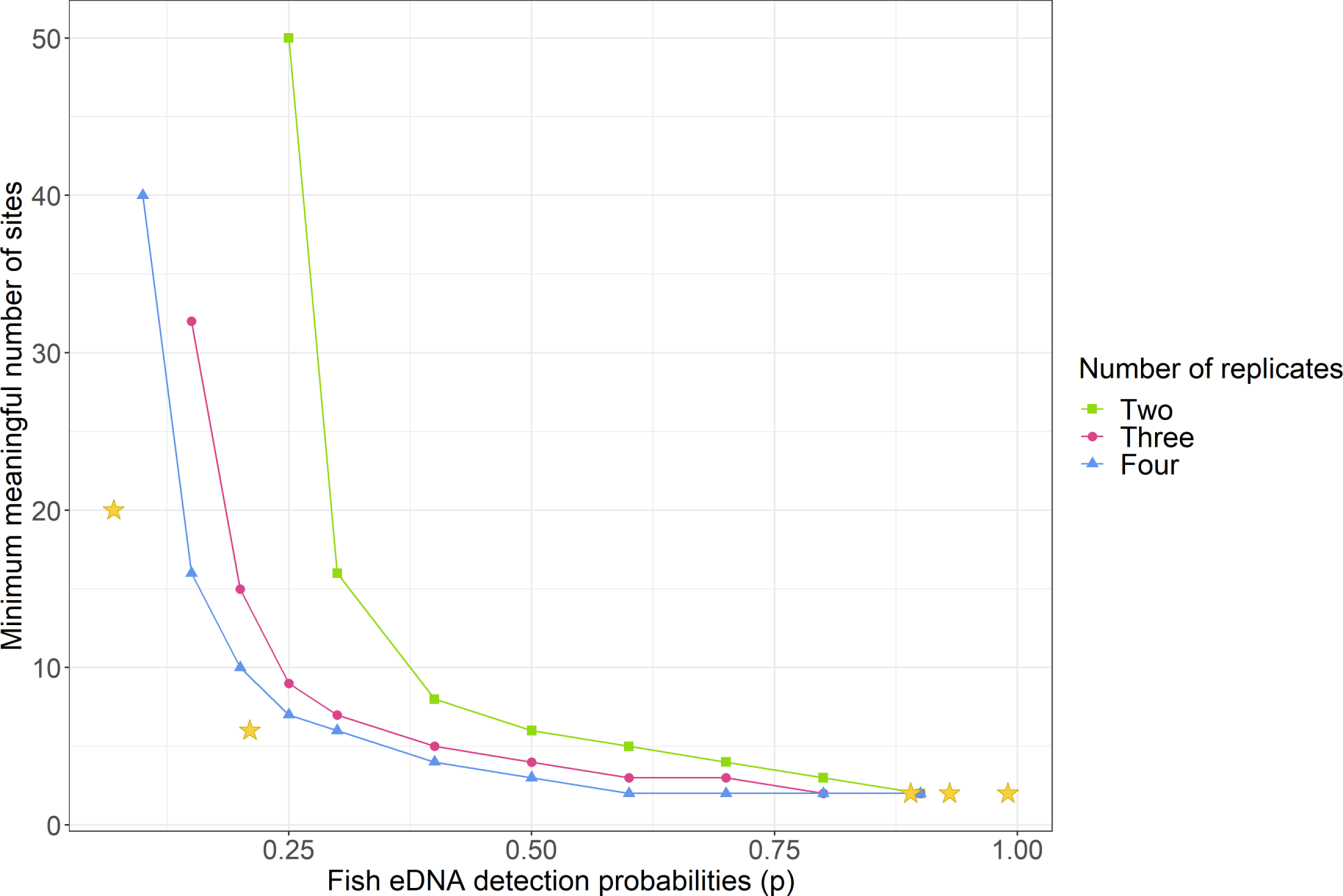
Comparison of the minimum number of sites needed to detect environmental DNA depending on detection probabilities and number of replicates. These simulations were based on the assumption that eDNA is present throughout the lake (Ψ = 1). The stars indicate where the study lakes fit according to the simulations: from left to right, Lake Tomarata water samples (*p* = 0.07, 20 sites), Lake Tomarata sediment samples (*p* = 0.21, 6 sites needed), Lake Waitawa perch eDNA water samples (*p* = 0.89, 2 sites needed), Lake Waitawa rudd eDNA water samples (*p* = 0.93, 2 sites needed), Lake Pounui sediment samples (*p* = 0.99, 2 sites needed).

To estimate the minimum required sampling effort, *i.e.,* the number of sampling sites and replicates at each site, for different probabilities of detection, simulations were run assuming fish eDNA is present and consistent across the lakes (Ψ = 1), for two, three and four replicates per site, and for detection probabilities of 0.1 to 0.9, (Fig. 4). Design estimates showed that adding more replicates decreased the number of sites needed for the same detection probability when *p* < 0.9, however for 0.3 ≤*p* ≤ 0.9 having three or four replicates made little difference.

The occupancy modelling calculated that the worst detection probability (p) for both methods were for rudd eDNA in Lake Tomarata (Table 3). Therefore, in a lake with low species abundance, to sample as few sites as possible and just by considering the detection probabilities of this study (at worst *p* = 0.21 for sediment and *p* = 0.07 for water, Lake Tomarata), the simulations indicated that at least six sites and five replicates (∼3 g) per site were needed to reliably detect fish using eDNA approaches in sediment samples (Table S9) and twenty sites with eight replicates (∼500 mL) per site were needed for water samples (Table S10).

Since the detection probabilities were very high for perch and rudd eDNA in Lake Pounui and Lake Waitawa, the simulations calculated that only two sites and two replicates per site were sufficient for reliable detection (Tables S11–S13; illustrated by a star in Fig. 4). The ideal sample type and location were suggested based on the occupancy model results (Table 3): in Lake Pounui, surface sediment from two sites near the shores would yield the best results (two replicates per site), while in Lake Waitawa, surface water from two sites anywhere in the lake would work better for both species (two replicates per site).

## Discussion

Designing robust sampling strategies is critical when using molecular approaches for monitoring lakes, especially when these are aimed at detecting organisms that are present in low abundance. Our results demonstrate that perch and rudd eDNA is relatively homogeneously distributed in small shallow lakes, however, the optimal sample type (sediment *versus* water) and location (near shore or mid-lake) varies among lakes. It’s likely that multiple factors, not all of which were explored in this study, account for this variability. These results, in concert with data from other studies, highlight the potential of using molecular techniques that target eDNA for detecting and monitoring fish in small shallow lakes.

### Distribution of fish environmental DNA in lakes

As hypothesized, in the present study fish eDNA was mostly homogeneously distributed across lakes regardless of the species. Similar results were obtained in a study targeting eDNA of the great crested newt in water and sediment samples from ponds, where their DNA was present in all samples collected (Buxton, Groombridge & Griffiths, 2018). Given the motile nature of these species and the high likelihood that they are constantly shedding some DNA, a homogeneous distribution is expected in these small systems. Lake Tomarata was the only lake where rudd eDNA (16S rDNA) was not homogeneously distributed, and instead only found at half of the sites. This is likely attributable to low population density, with only small numbers of this species thought to occur in this lake (Drake, Kelly & Schallenberg, 2011). Previous studies have also found a relationship between low quantitative PCR signal and low population density (Weldon et al., 2020).

### The effect of fish ecology on environmental DNA detection

The data contradicted our hypothesis that the best sample type would vary depending on fish ecology (benthic *versus* pelagic). The best sample type for perch and rudd in Lake Waitawa was water samples, while the best sample type for perch and rudd in Lakes Pounui and Tomarata was surface sediment. It was surprising that, despite being a pelagic fish, perch DNA was better detected in sediment samples. This may be due to the small and shallow nature of the lakes sampled, leading to quick deposition of DNA in the sediment. The higher detection rates of rudd in water samples (Lake Waitawa) were also surprising and may be due to sediment geochemistry in this lake (more in the following subsection).

The similar habitat preferences of perch and rudd may also have impacted detection, since both species prefer warm waters and their juveniles are known to cluster near the shores. This suggests our sampling approach was already adapted to the target species, and the study lakes may not have been deep enough to see significant differences in near-shore *versus* mid-lake sites. Lake Waitawa and Tomarata are quite shallow (max. depth around 5 m) therefore it is unlikely that perch and rudd would avoid the deepest sites. In comparison, Lake Pounui is around 10 m deep at its maximum, and near-shore sites yielded higher detection of perch eDNA compared to mid-lake sites. More research is needed to separate the influence of fish ecology compared to lake hydrology, since most findings are unconclusive so far. For example, studies in shallow systems have found higher detection of the greater crested newt DNA in water compared to sediment samples (Buxton, Groombridge & Griffiths, 2018), and more vertebrate species in water samples of shallow sites in Lake Bacalar (Mexico) compared to sediment (Valdez-Moreno et al., 2019). Conversely, big headed carp eDNA detection was much higher in pond and river sediment compared to water samples (Turner, Uy & Everhart, 2015). Studies in deeper systems have found more logical results, with a better detection of lake trout and char eDNA below the thermocline, which fits the ecology of these fish (Klobucar, Rodgers & Budy, 2017; Littlefair et al., 2021).

Perch eDNA was absent in the mid-lake water samples of Lake Pounui but was detected in all mid-lake sediment samples. The lack of perch DNA in the water samples does suggest that the populations of perch in Lake Pounui primarily inhabit the near-shore zone, which is corroborated by a previous study (Jellyman, 1980). The depocenter of lakes is known to be a site where sediment and other compounds accumulate and therefore selected for paleolimnology (Weisbrod et al., 2020). It is likely that this explains the detection of “cumulative” eDNA signal in these deeper samples. This could suggest positive results for future studies looking at fish sedimentary ancient DNA in shallow lakes, though for fish with a similar ecology to perch and rudd it may be preferable to take a sediment core in shallower waters.

### Variations in environmental DNA levels and detection

Lake Waitawa was the only lake out of the three where water samples provided better detection rates, for both perch and rudd DNA. The low levels and low detection rates in the sediment samples from Lake Waitawa could be related to sediment properties. Different sediment substrates bind eDNA with varying degrees of efficiency (Buxton, Groombridge & Griffiths, 2017). The sediment geochemistry data suggest that Lake Waitawa sediments have less available mineral particulate surfaces, which may reduce the possibility of DNA binding to sediment compared to Lakes Pounui and Tomarata. Lake Waitawa is the most eutrophic lake included in this study and experiences heavy cyanobacterial blooms every summer. This likely leads to overall low redox conditions on the surface sediment-water interface, which may also accelerate DNA decay (Sassoubre et al., 2016; Wei, Nakajima & Tobino, 2018). It is also the only lake of this study with houses on its shore, therefore contaminants from sewage waters may also alter sediment geochemistry. Unfortunately our analysis did not include tests for such contaminants.

Our results indicate that fish eDNA levels in shallow lakes may not always be related to population density. Catch data indicated perch density was higher than rudd in Lake Waitawa, and more perch were found in Lake Waitawa compared to Lake Pounui. However, overall the eDNA did not display the same trends. Weldon et al. (2020) found that eel eDNA levels in lakes (from water samples) only provide very coarse data on population density *i.e.,* low *vs* medium to high density, which may also be the case in our study. Although some catch data were available for each lake considered here, the sampling approaches applied during those studies prevent an accurate assessment of population densities. Additionally, the studies were undertaken multiple years prior to the eDNA surveys, preventing any further analysis of the relationship between eDNA concentrations and actual biomass.

Our data showed that detection rates across sample type were similar, but that fish eDNA concentration varied, which highlights the advantage of using both sediment and water sampling to understand patterns of fish occurrence and distribution in lakes. The presence of eDNA in surface sediment provides an indication that the target organism has been in the system sometime in the last weeks to months (*e.g.*, 132 days after carp removal—Turner, Uy & Everhart (2015), whereas DNA in water is more sensitive to decay and indicative of recent presence (up to ∼20 days Buxton, Groombridge & Griffiths, 2017; Troth et al., 2021). When sampling a new environment, we recommend using both water and sediment to maximize detection rates and obtain insights into whether any positive detections are likely due to the recent presence of an organism. This approach will also overcome challenges, such as those observed in the Lake Waitawa sediment samples, where lake-specific conditions, may inhibit detection.

Avoiding false negatives and false positives is extremely important in surveys targeting a given species (Ficetola et al., 2015; Smith & Goldberg, 2020; McClenaghan, Compson & Hajibabaei, 2020; Langlois et al., 2021). To reduce the chances of false positives from ddPCR, we followed a strict process during the interpretation of low-level positives. If only a single droplet was observed in a sample, it was run twice more and only accepted as a positive if a droplet was observed in two of the three replicates. We cannot rule out the possibility that the detection of perch in a single sample from Lake Tomarata (following the above protocols) is not due to contamination, although all negatives were clear. In cases where confirming the reliability of a low-level detection, or unexpected results is important, we recommend further testing of all available replicates and then returning to the site to undertake additional sampling using both eDNA and traditional approaches to confirm (or rebut) the detection.

### Considerations when designing a sampling program

Our findings may not be applicable to larger lakes. Studies on larger and deeper systems are relatively limited and given their more heterogeneous geomorphology, stronger currents, stratification, and greater dilution due to volume, they often require more complex sampling strategies. For example, studies have shown that stratification may impact the vertical distribution of eDNA in deeper lakes (Klobucar, Rodgers & Budy, 2017; Littlefair et al., 2021).

The simulations used to estimate the minimum reasonable sampling effort when developing a monitoring program were based on the lowest detection probabilities. They indicated that six sites, with five replicates were sufficient when taking sediment samples (*p* ≥ 0.21), and that a higher number of sites and replicates are suggested for water (since *p* ≥ 0.07), twenty sites and eight replicates. The ideal type of sample and ideal sampling location may vary depending on the lake, therefore we recommend taking both sample types across the lake, so that occupancy modelling and design simulations may then help determine which is better and how many sites/replicates are needed for a specific lake. The number of samples and replicates could be reduced for lakes with high naive fish eDNA occupancy such as Pounui and Waitawa (max. two sites and two replicates per site according to the simulations), but not when targeting species with low abundances such as rudd in Lake Tomarata. The high number of replicates required for water samples may become cost-prohibitive. To reduce the cost of analysis, tiered approaches have been suggested (Sham et al., 2002). This could involve pooling samples (*e.g.*, a portion of all water samples from a single lake) for an initial screening and if a positive signal is obtained then all replicates should be analyzed. One limitation of this approach is that weak signals are diluted, which may result in false negatives.

A further consideration that influences detection is sample size. In this study we extracted eDNA from c. 3 g of sediment and 500 mL of water. We initially tried with 0.25 g of sediment and detection rates were very low (sediments of Lake Pounui, Table S14). The DNA extraction method applied in this study has been shown to be effective on up to 10 g of sediment (Thomson-Laing et al., 2022). Extracting from larger sediment volumes may have improved detection rates, although it can also create other issues such as greater inhibition. Likewise, studies have found that increasing the volume of water sampled increases eDNA levels and detection rates (Sepulveda et al., 2019). The type of filter used also impacts detection (Hunter et al., 2019). During our study two of the three lakes were experiencing cyanobacterial blooms, which limited the volume of water that could be filtered, despite pre-filtration for the Lake Waitawa samples. In our study we filtered 1 liter of water and the filter was cut in half to avoid clogging the tubes used for extraction, which effectively meant that only 500 mL of water was analyzed. Extracting both halves (therefore 1 L) and combining the DNA extracts could potentially increase the sensitivity of our assay without filtering more water. Furthermore, increasing the pore size of the filter (*e.g.*, by using a nylon filter) would be another option (Zaiko et al., 2022) but would need optimization to ensure that DNA is not lost through the pores. The development of new techniques such as passive sampling provide new avenues that may overcome some of the limitation with sampling water and sediment samples (Kirtane, Atkinson & Sassoubre, 2020; Bessey et al., 2021; Verdier et al., 2022), but further research is required to determine their applicability for specific species.

## Conclusions

The results of this study demonstrate that perch and rudd eDNA can be detected in lake water or surface sediment. In general surface sediment samples had higher detection rates but there were differences among lakes which we attribute to factors such as sediment geochemistry. When initiating a sampling program, we recommend initially including both water and sediment samples. The data generated from the two sample types also provide complementary information on fish dynamics. The sediment samples give information on fish presence integrated over a longer time frame, whereas the water samples provide contemporary insights. Occupancy modelling undertaken in this study indicates that for both perch and rudd, sampling near the shores of the lake is similar if not better than sampling in deeper parts of the lake—a valuable finding given that this reduced the need for boats and more complex sampling requirements. Using the detection rate data generated in the present study, we predicted that at least six sites and five replicates per site would be needed to reliably detect fish eDNA in sediment samples, and twenty sites with eight replicates per site for water samples. The techniques used here could be applied to other fish species to aid in developing informed monitoring or surveillance programs.

##  Supplemental Information

10.7717/peerj.15210/supp-1 Supplemental Information 1 Supplementary material

10.7717/peerj.15210/supp-2 Supplemental Information 2 Fish eDNA levels and metadata for all samples Each row corresponds to a single replicate. Each replicate was sampled in one of three lakes (Pounui, Tomarata, or Waitawa), in one of fourteen sites, using one of two methods (water or sediment). Sampling metadata (site coordinates, location, sediment depths) is provided. Normalised ddPCR concentrations are the values used in the study. Raw DNA concentrations from droplet digital PCR are also provided (Conc.uL_Perch or Conc.uL_Rudd) as well as the confidence intervals calculated by the QuantaSoft software.

10.7717/peerj.15210/supp-3 Supplemental Information 3 Dilution series from positive extracts of perch and rudd used to calculate the Limit of Quantification Concentrations were obtained through droplet digital PCR. Code used for calculation available on GitHub

## Additional Information and Declarations

### Field Study Permissions

The following information was supplied relating to field study approvals (i.e., approving body and any reference numbers): All samples were collected under the specifications of Special Permit 651 from the New Zealand government agency Ministry for Primary Industries.

### Data Availability

The following information was supplied regarding data availability: The code is available at GitHub and Zenodo: https://github.com/MLysP/Spatial-survey-of-exotic-fish-using-eDNA. Picard, M. (2023). Script for optimal sample type and number vary in small shallow lakes when targeting non-native fish environmental DNA (v1.0.1). Zenodo. https://doi.org/10.5281/zenodo.7741480. The raw data is available in the Supplementary File.
