# Supplementary material for: Optimal sample type and number vary in small shallow lakes when targeting non-native fish environmental DNA
Source: PeerJ. 2023 May 2;11:e15210. doi: 10.7717/peerj.15210 (PMC10162041; doi:10.7717/peerj.15210)
Supplement: Supplemental Information 1 [file peerj-11-15210-s001.docx]

# **Supplementary material – Picard et al. (2023)**

# **Optimal sample type and number vary in small shallow lakes**

# **when targeting non-native fish environmental DNA**

**
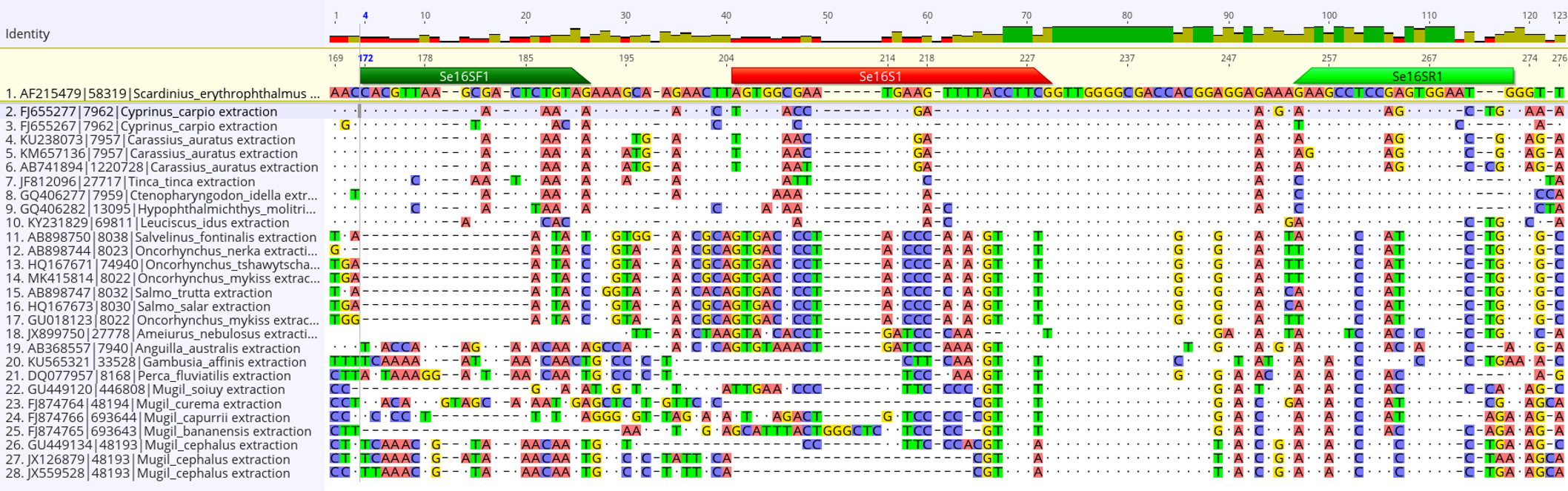
**

***Figure S1****: Selection of the rudd alignment and position of the primers and probe. The alignment was realised on 87 sequences, and identical sequences from the same species were removed for this figure. Rudd is set as the reference sequence compared to all other sequences. Dots indicate identical nucleotides at the same position as the reference sequence, while nucleotides are indicated and color-coded for all disagreements.*

**Table S1**: Details of the sampling sites.

| Lake | Sampling Date | Location | Site | Depth (m) | Easting | Northing |
| --- | --- | --- | --- | --- | --- | --- |
| Pounui | 4/12/2018 | Mid-lake | 1 | 6 | 175.1148 | -41.3445 |
|  |  |  | 2 | 9.1 | 175.1124 | -41.3445 |
|  |  |  | 3 | 9.8 | 175.1086 | -41.3437 |
|  |  |  | 4 | 7.3 | 175.1172 | -41.3452 |
|  |  |  | 5 | 7.5 | 175.1133 | -41.3465 |
|  |  |  | 6 | 8.4 | 175.1154 | -41.3462 |
|  |  |  | 7 | 5.3 | 175.1118 | -41.3471 |
|  |  | Near-shore | 8 | 2.1 | 175.1181 | -41.3431 |
|  |  |  | 9 | 2.5 | 175.1127 | -41.3423 |
|  |  |  | 10 | 1.4 | 175.1097 | -41.3431 |
|  |  |  | 11 | 1.8 | 175.1068 | -41.3431 |
|  |  |  | 12 | 2 | 175.1094 | -41.3474 |
|  |  |  | 13 | 2.1 | 175.1137 | -41.3472 |
|  |  |  | 14 | 1.5 | 175.1184 | -41.3456 |
| Tomarata | 18/11/2018 | Near-shore | 1 | 2.8 | 174.6474 | -36.19243 |
|  |  |  | 2 | 2.7 | 174.6482 | -36.1939 |
|  |  |  | 3 | 3.7 | 174.6504 | -36.19547 |
|  |  |  | 4 | 2.9 | 174.6531 | -36.19456 |
|  |  |  | 5 | 3.1 | 174.6515 | -36.1927 |
|  |  |  | 6 | 5.1 | 174.6503 | -36.19234 |
|  |  |  | 7 | 4 | 174.6482 | -36.19171 |
|  |  | Mid-lake | 8 | 5.4 | 174.648 | -36.19256 |
|  |  |  | 9 | 5.1 | 174.6487 | -36.19349 |
|  |  |  | 10 | 4.7 | 174.6503 | -36.19442 |
|  |  |  | 11 | 4.7 | 174.6514 | -36.19446 |
|  |  |  | 12 | 4.8 | 174.6505 | -36.19347 |
|  |  |  | 13 | 5.6 | 174.6488 | -36.19244 |
|  |  |  | 14 | 5.2 | 174.6494 | -36.19338 |
| Waitawa | 13/10/2019 | Mid-lake | 1 | 6.5 | 178.3612 | -54.89617 |
|  |  |  | 2 | 6.5 | 178.3557 | -54.89575 |
|  |  |  | 3 | 6.8 | 178.346 | -54.89537 |
|  |  |  | 4 | 6.9 | 178.335 | -54.89545 |
|  |  |  | 5 | 7 | 178.3261 | -54.89558 |
|  |  |  | 6 | 4.5 | 178.318 | -54.89443 |
|  |  |  | 7 | 6.5 | 178.332 | -54.8938 |
|  |  | Near-shore | 8 | 2.5 | 178.3476 | -54.89295 |
|  |  |  | 9 | 1.8 | 178.3125 | -54.89401 |
|  |  |  | 10 | 2.9 | 178.3227 | -54.89625 |
|  |  |  | 11 | 2.7 | 178.3633 | -54.89786 |
|  |  |  | 12 | 2.6 | 178.3671 | -54.89863 |
|  |  |  | 13 | 2.2 | 178.3772 | -54.89609 |
|  |  |  | 14 | 2.7 | 178.3874 | -54.89647 |

**Table S2**: List of fish species against which the rudd assay has been tested. The method column indicates how the specificity was checked: qPCR on tissue = fish tissue extracts were directly tested on a qPCR machine; metabarcoding and qPCR = samples of known species composition (metabarcoding) were also tested with the rudd assay and returned a negative signal. The full list of species tested in the metabarcoding samples is in Table S3.

| **Method** | **Fish common name** | **Fish latin name** |
| --- | --- | --- |
| qPCR on tissue | Brown bullhead catfish | *Ameiurus nebulosus* |
|  | Goldfish | *Carassius auratus* |
|  | Koi carp | *Cyprinus rubrofiscus* |
|  | Gambusia | *Gambusia affinis* |
|  | European perch | *Perca fluviatilis* |
|  | Tench | *Tinca tinca* |
| Metabarcoding & qPCR | Brown bullhead catfish | *Ameiurus nebulosus* |
|  | Short-finned eel | *Anguilla australis* |
|  | Goldfish | *Carassius auratus* |
|  | Gambusia | *Gambusia affinis* |
|  | Skipjack tuna | *Katsuwonus pelamis* |
|  | Rainbow trout | *Oncorhynchus mykiss* |
|  | Smelt | *Retropinna retropinna* |
|  | Brown trout | *Salmo trutta* |
|  | Tench | *Tinca tinca* |

**Table S3**: Details of the metabarcoding samples on which the rudd assay was tested. Fish amplicon sequence variants were summed by species.

| **Collection Date** | ***Latitude*** | ***Longitude*** | | ***Volume filtered (mL)*** | ***Rudd qPCR*** | ***Fish detected using metabarcoding (number of reads)*** |
| --- | --- | --- | --- | --- | --- | --- |
| 27/11/2019 | -38.0317 | 176.3514 | 300 | | Negative | Ameiurus nebulosus (58); Carassius auratus (841); Retropinna retropinna(30); Gobiomorphus (2309); Gambusia affinis (14) Ameiurus nebulosus (99); Carassius auratus (1748); Retropinna retropinna(93); Gobiomorphus (932); Gambusia affinis (36) Ameiurus nebulosus (220); Katsuwonus pelamis (31); Oncorhynchus mykiss(322); Oncorhynchus mykiss (547); Salmo trutta (53); Retropinna retropinna (119); Retropinna retropinna (6116); Gobiomorphus (69); Gobiomorphus (20463); Gambusia affinis (126); |
| 27/11/2019 | -38.0317 | 176.3514 | 240 | | Negative | Ameiurus nebulosus (99); Carassius auratus (1748); Retropinna retropinna(93); Gobiomorphus (932); Gambusia affinis (36) Ameiurus nebulosus (220); Katsuwonus pelamis (31); Oncorhynchus mykiss(322); Oncorhynchus mykiss (547); Salmo trutta (53); Retropinna retropinna (119); Retropinna retropinna (6116); Gobiomorphus (69); Gobiomorphus (20463); Gambusia affinis (126); Scardinius erythrophthalmus (82); Anguilla australis (1151); Anguilla dieffenbachii(383); Anguilla australis (786); Perca fluviatilis (374); Gobiomorphus (6947)); |
| 28/08/2019 | -38.1286 | 176.2501 | 130 | | Negative | Ameiurus nebulosus (220); Katsuwonus pelamis (31); Oncorhynchus mykiss(322); Oncorhynchus mykiss (547); Salmo trutta (53); Retropinna retropinna (119); Retropinna retropinna (6116); Gobiomorphus (69); Gobiomorphus (20463); Gambusia affinis (126); Scardinius erythrophthalmus (82); Anguilla australis (1151); Anguilla dieffenbachii(383); Anguilla australis (786); Perca fluviatilis (374); Gobiomorphus (6947)); Scardinius erythrophthalmus (52); Anguilla australis (450); Anguilla dieffenbachii(21); Perca fluviatilis (80); Gobiomorphus (1183) |
| 16/12/2019 | -39.0113 | 174.2434 | 300 | | Positive | Scardinius erythrophthalmus (82); Anguilla australis (1151); Anguilla dieffenbachii (383); Anguilla australis (786); Perca fluviatilis (374); Gobiomorphus (6947)); Scardinius erythrophthalmus (52); Anguilla australis (450); Anguilla dieffenbachii (21); Perca fluviatilis (80); Gobiomorphus (1183) Carassius auratus (52); Scardinius erythrophthalmus (94); Anguilla australis(193); Perca fluviatilis (108); Gobiomorphus (22) |
| 16/12/2019 | -39.0113 | 174.2434 | 480 | | Positive | Scardinius erythrophthalmus (52); Anguilla australis (450); Anguilla dieffenbachii (21); Perca fluviatilis (80); Gobiomorphus (1183) Carassius auratus (52); Scardinius erythrophthalmus (94); Anguilla australis (193); Perca fluviatilis (108); Gobiomorphus (22) Scardinius erythrophthalmus (26); Anguilla australis (215); Perca fluviatilis (421); Gobiomorphus (517) |
| 16/12/2019 | -39.0113 | 174.2434 | 480 | | Positive | Carassius auratus (52); Scardinius erythrophthalmus (94); Anguilla australis (193); Perca fluviatilis (108); Gobiomorphus (22) Scardinius erythrophthalmus (26); Anguilla australis (215); Perca fluviatilis (421); Gobiomorphus (517) Carassius auratus (40); Scardinius erythrophthalmus (13); Anguilla australis (72); Anguilla australis (296); Perca fluviatilis (28); Gobiomorphus (56) |
| 16/12/2019 | -39.0113 | 174.2434 | 600 | | Positive | Scardinius erythrophthalmus (26); Anguilla australis (215); Perca fluviatilis(421); Gobiomorphus (517) Carassius auratus (40); Scardinius erythrophthalmus (13); Anguilla australis(72); Anguilla australis (296); Perca fluviatilis (28); Gobiomorphus (56) Anguilla australis (45); Scardinius erythrophthalmus (56); Cyprinus rubrofuscus (908); Carassius auratus (729); Carassius auratus (19841); Ameiurus nebulosus (13); Cyprinus rubrofuscus (4008); Carassius auratus(39244); Scardinius erythrophthalmus (55); Ameiurus nebulosus (16); Anguilla australis (46); Gobiomorphus (26) |
| 16/12/2019 | -39.0113 | 174.2434 | 480 | | Positive | Carassius auratus (40); Scardinius erythrophthalmus (13); Anguilla australis(72); Anguilla australis (296); Perca fluviatilis (28); Gobiomorphus (56) Anguilla australis (45); Scardinius erythrophthalmus (56); Cyprinus rubrofuscus(908); Carassius auratus (729); Carassius auratus (19841); Ameiurus nebulosus (13); Cyprinus rubrofuscus (4008); Carassius auratus(39244); Scardinius erythrophthalmus (55); Ameiurus nebulosus (16); Anguilla australis (46); Gobiomorphus (26)Gambusia affinis (100); Gambusia affinis (100); Anguilla australis(39); Scardinius erythrophthalmus (548); Carassius auratus (4352); Ameiurus nebulosus (380); Carassius auratus (25701); Scardinius erythrophthalmus(2827); Ameiurus nebulosus (157); Anguilla australis (59); Gambusia affinis (235); |
| 22/11/2019 | -37.9448 | 175.2821 | 60 | | Positive | Anguilla australis (45); Scardinius erythrophthalmus (56); Cyprinus rubrofuscus (908); Carassius auratus (729); Carassius auratus (19841); Ameiurus nebulosus (13); Cyprinus rubrofuscus (4008); Carassius auratus(39244); Scardinius erythrophthalmus (55); Ameiurus nebulosus (16); Anguilla australis (46); Gobiomorphus (26)Gambusia affinis (100); Gambusia affinis (100); Anguilla australis(39); Scardinius erythrophthalmus (548); Carassius auratus (4352); Ameiurus nebulosus (380); Carassius auratus (25701); Scardinius erythrophthalmus(2827); Ameiurus nebulosus (157); Anguilla australis (59); Gambusia affinis (235); Anguilla australis (4); Carassius auratus (304); Carassius auratus(1904); Ameiurus nebulosus (151); Cyprinus rubrofuscus (41807); Carassius auratus (36974); Scardinius erythrophthalmus (202); Retropinna retropinna(2); Ameiurus nebulosus (20); Gobiomorphus (88); Gambusia affinis (9); |
| 22/11/2019 | -37.9279 | 175.2883 | 110 | | Positive | Gambusia affinis (100); Gambusia affinis (100); Anguilla australis(39); Scardinius erythrophthalmus (548); Carassius auratus (4352); Ameiurus nebulosus (380); Carassius auratus (25701); Scardinius erythrophthalmus(2827); Ameiurus nebulosus (157); Anguilla australis (59); Gambusia affinis (235); Anguilla australis (4); Carassius auratus (304); Carassius auratus(1904); Ameiurus nebulosus (151); Cyprinus rubrofuscus (41807); Carassius auratus (36974); Scardinius erythrophthalmus (202); Retropinna retropinna(2); Ameiurus nebulosus (20); Gobiomorphus (88); Gambusia affinis (9); Scardinius erythrophthalmus (18); Carassius auratus (440); Ameiurus nebulosus(101); Carassius auratus (2960); Scardinius erythrophthalmus (130); Ameiurus nebulosus (11); Anguilla australis (4); Gambusia affinis(5); |
| 22/11/2019 | -37.9606 | 175.2949 | 60 | | Positive | Anguilla australis (4); Carassius auratus (304); Carassius auratus(1904); Ameiurus nebulosus (151); Cyprinus rubrofuscus (41807); Carassius auratus (36974); Scardinius erythrophthalmus (202); Retropinna retropinna(2); Ameiurus nebulosus (20); Gobiomorphus (88); Gambusia affinis (9); Scardinius erythrophthalmus (18); Carassius auratus (440); Ameiurus nebulosus(101); Carassius auratus (2960); Scardinius erythrophthalmus (130); Ameiurus nebulosus (11); Anguilla australis (4); Gambusia affinis(5); Gambusia affinis (9); Gambusia affinis (9); Anguilla australis(40); Scardinius erythrophthalmus (54); Cyprinus rubrofuscus (89); Carassius auratus (15332); Ameiurus nebulosus (37); Carassius auratus(331); Cyprinus rubrofuscus (1502); Carassius auratus (107434); Scardinius erythrophthalmus (289); Anguilla australis (297); Gambusia affinis (83); |
| 22/11/2019 | -37.9258 | 175.2917 | 75 | | Positive | Scardinius erythrophthalmus (18); Carassius auratus (440); Ameiurus nebulosus(101); Carassius auratus (2960); Scardinius erythrophthalmus (130); Ameiurus nebulosus (11); Anguilla australis (4); Gambusia affinis(5); Gambusia affinis (9); Gambusia affinis (9); Anguilla australis(40); Scardinius erythrophthalmus (54); Cyprinus rubrofuscus (89); Carassius auratus (15332); Ameiurus nebulosus (37); Carassius auratus(331); Cyprinus rubrofuscus (1502); Carassius auratus (107434); Scardinius erythrophthalmus (289); Anguilla australis (297); Gambusia affinis (83); Gambusia affinis (14144); Gobiomorphus (194); Gobiomorphus (22142); Gobiomorphus (578); Anguilla australis (2727); Carassius auratus (4618); Ameiurus nebulosus (19); Carassius auratus(393); Retropinnidae (424); Ameiurus nebulosus (830); Anguilla australis (78); Gobiomorphus (857); Gobiomorphus 293); Gambusia affinis (801); |
| 22/11/2019 | -37.9548 | 175.2938 | 70 | | Positive | Gambusia affinis (9); Gambusia affinis (9); Anguilla australis(40); Scardinius erythrophthalmus (54); Cyprinus rubrofuscus (89); Carassius auratus (15332); Ameiurus nebulosus (37); Carassius auratus(331); Cyprinus rubrofuscus (1502); Carassius auratus (107434); Scardinius erythrophthalmus (289); Anguilla australis (297); Gambusia affinis (83); Gambusia affinis (14144); Gobiomorphus (194); Gobiomorphus (22142); Gobiomorphus (578); Anguilla australis (2727); Carassius auratus (4618); Ameiurus nebulosus (19); Carassius auratus(393); Retropinnidae (424); Ameiurus nebulosus (830); Anguilla australis (78); Gobiomorphus (857); Gobiomorphus 293); Gambusia affinis (801); Cypriniformes (90); Cyprinoidei (128); Carassius auratus(233); Carassius auratus (44171); Cyprinoidei (19); Cypriniformes (267); Carassius auratus (1260); Cyprinidae (46); Carassius auratus (1596); Scardinius erythrophthalmus (125); Ameiurus nebulosus (2963) |
| 22/11/2019 | -37.9497 | 175.3203 | 360 | | Negative | Gambusia affinis (14144); Gobiomorphus (194); Gobiomorphus (22142); Gobiomorphus (578); Anguilla australis (2727); Carassius auratus (4618); Ameiurus nebulosus (19); Carassius auratus(393); Retropinnidae (424); Ameiurus nebulosus (830); Anguilla australis (78); Gobiomorphus (857); Gobiomorphus 293); Gambusia affinis (801); Cypriniformes (90); Cyprinoidei (128); Carassius auratus(233); Carassius auratus (44171); Cyprinoidei (19); Cypriniformes (267); Carassius auratus (1260); Cyprinidae (46); Carassius auratus (1596); Scardinius erythrophthalmus (125); Ameiurus nebulosus (2963) Gambusia affinis (977); Gobiomorphus (11028); Gobiomorphus (297); Anguilla australis (545); Carassius auratus (5618); Ameiurus nebulosus (531); Carassius auratus (4762); Retropinnidae (348); Retropinnidae (416); Ameiurus nebulosus (476); Anguilla australis (334); Anguilla australis (110); Retropinna retropinna (799); Gobiomorphus (5969); Gobiomorphus (3558); Gambusia affinis (866); |
| 22/11/2019 | -37.9274 | 175.2932 | 88 | | Positive | Cypriniformes (90); Cyprinoidei (128); Carassius auratus(233); Carassius auratus (44171); Cyprinoidei (19); Cypriniformes (267); Carassius auratus (1260); Cyprinidae (46); Carassius auratus (1596); Scardinius erythrophthalmus (125); Ameiurus nebulosus (2963) Gambusia affinis (977); Gobiomorphus (11028); Gobiomorphus (297); Anguilla australis (545); Carassius auratus (5618); Ameiurus nebulosus (531); Carassius auratus (4762); Retropinnidae (348); Retropinnidae (416); Ameiurus nebulosus (476); Anguilla australis (334); Anguilla australis (110); Retropinna retropinna (799); Gobiomorphus (5969); Gobiomorphus (3558); Gambusia affinis (866); Gambusia affinis (236); Gambusia affinis (236); Gobiomorphus (2394); Anguilla australis (110); Scardinius erythrophthalmus (7); Carassius auratus (830); Ameiurus nebulosus (302); Carassius auratus(3425); Retropinnidae (73); Ameiurus nebulosus (98); Anguilla australis (190); Retropinna retropinna (1175); Gobiomorphus (4746); Gobiomorphus (5492); Gambusia affinis (1053) |
| 22/11/2019 | -37.9511 | 175.3206 | 390 | | Negative | Gambusia affinis (977); Gobiomorphus (11028); Gobiomorphus (297); Anguilla australis (545); Carassius auratus (5618); Ameiurus nebulosus (531); Carassius auratus (4762); Retropinnidae (348); Retropinnidae (416); Ameiurus nebulosus (476); Anguilla australis (334); Anguilla australis (110); Retropinna retropinna (799); Gobiomorphus (5969); Gobiomorphus (3558); Gambusia affinis (866); Gambusia affinis (236); Gambusia affinis (236); Gobiomorphus (2394); Anguilla australis (110); Scardinius erythrophthalmus (7); Carassius auratus (830); Ameiurus nebulosus (302); Carassius auratus(3425); Retropinnidae (73); Ameiurus nebulosus (98); Anguilla australis (190); Retropinna retropinna (1175); Gobiomorphus (4746); Gobiomorphus (5492); Gambusia affinis (1053)Gambusia affinis (13); Galaxias maculatus (28); Mugil cephalus(31); Mugil cephalus (68); Gobiomorphus (482); Cheimarrichthys fosteri (50); Salmo trutta (16); Anguilla australis(217); Scardinius erythrophthalmus (65); Cyprinus rubrofuscus (324); Carassius auratus (40); Cheimarrichthys fosteri (195); Cyprinus rubrofuscus (1326); Carassius auratus (184); Scardinius erythrophthalmus (407); Retropinnidae (229); Retropinna retropinna (214); Ameiurusnebulosus17); Anguilla reinhardtii (43); Anguilla australis (624); Anguilla dieffenbachii (168 |
| 22/11/2019 | -37.9496 | 175.3202 | 480 | | Negative | Gambusia affinis (236); Gambusia affinis (236); Gobiomorphus (2394); Anguilla australis (110); Scardinius erythrophthalmus (7); Carassius auratus (830); Ameiurus nebulosus (302); Carassius auratus(3425); Retropinnidae (73); Ameiurus nebulosus (98); Anguilla australis (190); Retropinna retropinna (1175); Gobiomorphus (4746); Gobiomorphus (5492); Gambusia affinis (1053)Gambusia affinis (13); Galaxias maculatus (28); Mugil cephalus(31); Mugil cephalus (68); Gobiomorphus (482); Cheimarrichthys fosteri (50); Salmo trutta (16); Anguilla australis(217); Scardinius erythrophthalmus (65); Cyprinus rubrofuscus (324); Carassius auratus (40); Cheimarrichthys fosteri (195); Cyprinus rubrofuscus (1326); Carassius auratus (184); Scardinius erythrophthalmus (407); Retropinnidae (229); Retropinna retropinna (214); Ameiurusnebulosus17); Anguilla reinhardtii (43); Anguilla australis (624); Anguilla dieffenbachii (168Mugil cephalus (42); Gobiomorphus (408); Cheimarrichthys fosteri(110); Anguilla australis (94); Scardinius erythrophthalmus (23); Cyprinus rubrofuscus (330); Carassius auratus (33); Cheimarrichthys fosteri(262); Cyprinus rubrofuscus (1619); Carassius auratus (304); Scardinius erythrophthalmus (119); Retropinnidae (120); Retropinna retropinna (154); Anguilla australis (463); Anguilla dieffenbachii (70); Salmo trutta (74); Retropinna retropinna (1500); Retropinnaretropinna1524); Retropinna retropinna (205); Gobiomorphus (2205); Gobiomorphus (82 |
| 20/02/2020 | -37.7927 | 175.2913 | 500 | | Positive | Gambusia affinis (13); Galaxias maculatus (28); Mugil cephalus(31); Mugil cephalus (68); Gobiomorphus (482); Cheimarrichthys fosteri (50); Salmo trutta (16); Anguilla australis(217); Scardinius erythrophthalmus (65); Cyprinus rubrofuscus (324); Carassius auratus (40); Cheimarrichthys fosteri (195); Cyprinus rubrofuscus (1326); Carassius auratus (184); Scardinius erythrophthalmus (407); Retropinnidae (229); Retropinna retropinna (214); Ameiurusnebulosus17); Anguilla reinhardtii (43); Anguilla australis (624); Anguilla dieffenbachii (168); Salmo trutta (35); Retropinna retropinna (745); Retropinna retropinna (1002); Gobiomorphus (949); Gobiomorphus (264); Gambusia Mugil cephalus (42); Gobiomorphus (408); Cheimarrichthys fosteri(110); Anguilla australis (94); Scardinius erythrophthalmus (23); Cyprinus rubrofuscus (330); Carassius auratus (33); Cheimarrichthys fosteri(262); Cyprinus rubrofuscus (1619); Carassius auratus (304); Scardinius erythrophthalmus (119); Retropinnidae (120); Retropinna retropinna (154); Anguilla australis (463); Anguilla dieffenbachii (70); Salmo trutta (74); Retropinna retropinna (1500); Retropinnaretropinna1524); Retropinna retropinna (205); Gobiomorphus (2205); Gobiomorphus (82); Mugil cephalus (291); Galaxias maculatus (70); Galaxias maculatus (67); Galaxias maculatus (55); Galaxias maculatus (163); Galaxias Mugil cephalus (181); Mugil cephalus (251); Gobiomorphus (2167); Gobiomorphus cotidianus (81); Cheimarrichthys fosteri (493); Salmo trutta (148); Anguilla australis (551); Anguilla (58); Cyprinus rubrofuscus (1525); Carassius auratus (281); Cheimarrichthys fosteri (23); Cyprinus rubrofuscus (570); Carassius auratus (48); Scardinius erythrophthalmus (30); Retropinnidae (549); Retropinna retropinna (1035); Anguilla australis (195); Salmotrutta72); Retropinna retropinna (542); Retropinna retropinna (457); Gobiomorphus (650); Gobiomorphus (131); Mugil cephalus (107); Mugil cephalus (25); Galaxias maculatus (16); |
| 19/02/2020 | -37.7927 | 175.2913 | 500 | | Positive | Mugil cephalus (42); Gobiomorphus (408); Cheimarrichthys fosteri(110); Anguilla australis (94); Scardinius erythrophthalmus (23); Cyprinus rubrofuscus (330); Carassius auratus (33); Cheimarrichthys fosteri(262); Cyprinus rubrofuscus (1619); Carassius auratus (304); Scardinius erythrophthalmus (119); Retropinnidae (120); Retropinna retropinna (154); Anguilla australis (463); Anguilla dieffenbachii (70); Salmo trutta (74); Retropinna retropinna (1500); Retropinnaretropinna1524); Mugil cephalus (181); Mugil cephalus (251); Gobiomorphus (2167); Gobiomorphus cotidianus (81); Cheimarrichthys fosteri (493); Salmo trutta (148); Anguilla australis (551); Anguilla (58); Cyprinus rubrofuscus (1525); Carassius auratus (281); Cheimarrichthys fosteri (23); Cyprinus rubrofuscus (570); Carassius auratus (48); Scardinius erythrophthalmus (30); Retropinnidae (549); Retropinna retropinna (1035); Anguilla australis (195); Salmotrutta72); Carassius auratus (5397); Tinca tinca (4278); Anguilla australis(533); Anguilla australis (213) |
| 20/02/2020 | -37.7927 | 175.2913 | 500 | | Positive | Mugil cephalus (181); Mugil cephalus (251); Gobiomorphus (2167); Gobiomorphus cotidianus (81); Cheimarrichthys fosteri (493); Salmo trutta (148); Anguilla australis (551); Anguilla (58); Cyprinus rubrofuscus (1525); Carassius auratus (281); Cheimarrichthys fosteri (23); Cyprinus rubrofuscus (570); Carassius auratus (48); Scardinius erythrophthalmus (30); Retropinnidae (549); Retropinna retropinna (1035); Anguilla australis (195); Carassius auratus (5397); Tinca tinca (4278); Anguilla australis(533); Anguilla australis (213) |
| 11/02/2020 | -41.1596 | 172.9975 | 600 | | Negative | Carassius auratus (5397); Tinca tinca (4278); Anguilla australis(533); Anguilla australis (213) |


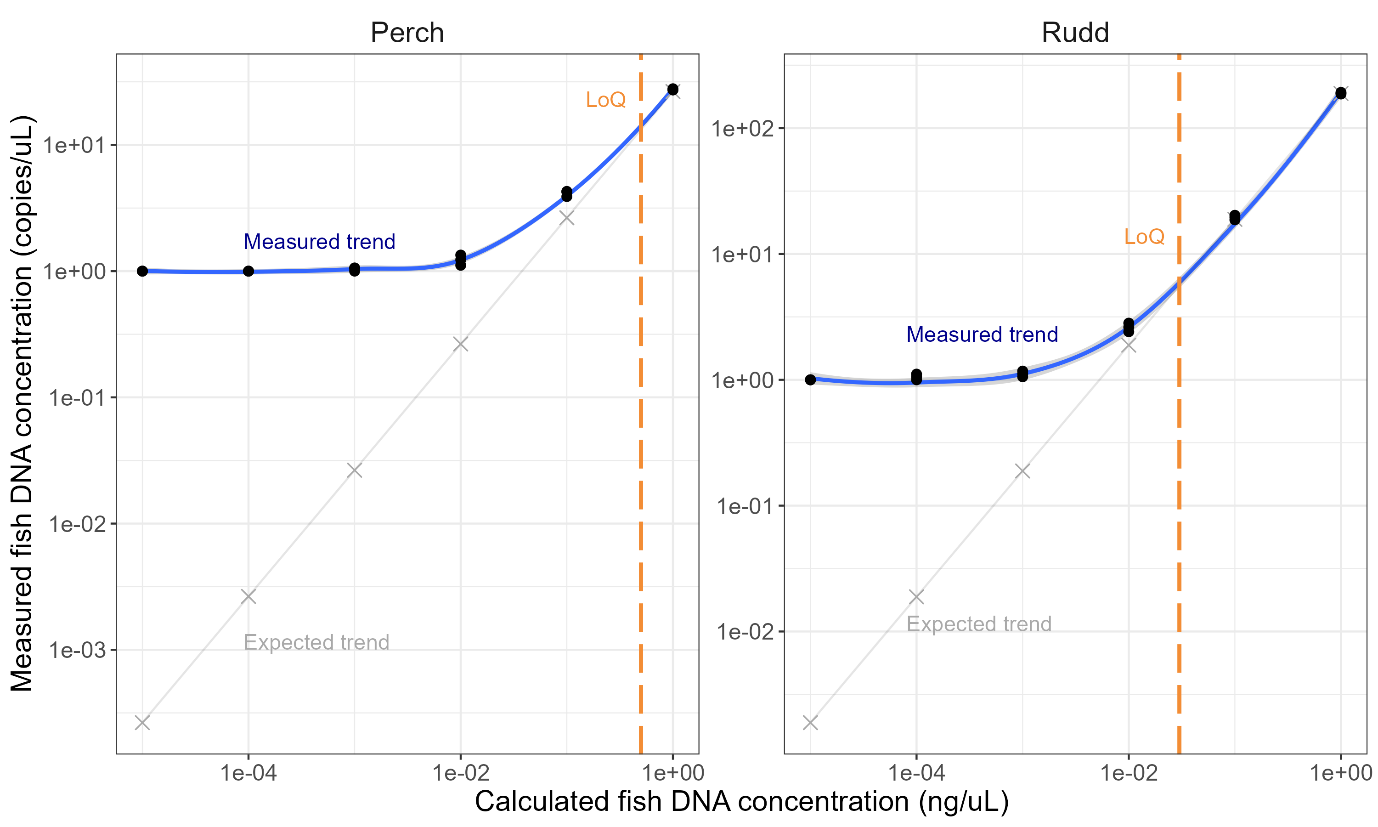


**Figure S2**: Limit of quantification (LoQ) obtained by measuring diluted perch and rudd DNA extracts with the perch-rudd duplex assay on a droplet digital PCR system. Raw extracts were diluted to obtain a starting concentration of 1 ng/ µL, and further diluted (0.1 ng/ µL, 0.01 ng/ µL, 0.001 ng/ µL, 0.0001 ng/ µL, 0.00001 ng/ µL). Dilutions were run in triplicate. Calculated concentrations were plotted against measured concentrations (log scale for both axis), with the grey line representing the expected trend, and the blue line representing the measured trend using a loess function. The LoQ was set as the point where the measured trend diverged from the expected trend: 13 copies/µL for perch and 5 copies/µL for rudd. Measured concentrations are in copies/µL per ddPCR sample analysed.

**Table S4**: Surface sediment geochemistry (single spot sampling from the deepest part of each lake).

|  | **Lake Pounui** | **Lake Tomarata** | **Lake Waitawa** |
| --- | --- | --- | --- |
| Date Surveyed | 25/09/2018 | 14/11/2018 | 24/09/2018 |
| Dry Weight (kg.m^-3^) | 128.9 | 205.4 | 21.4 |
| Total Produced Water (%) | 86.8 | 86.7 | 97.9 |
| Ash (g/100g dry wt) | 85.1 | 74.5 | 66.1 |
| Organic Matter (g/100g dry wt) | 14.9 | 25.5 | 33.9 |
| Organic Matter / Ash | 0.18 | 0.34 | 0.51 |
| Carbonates (g/100g dry wt) | 1.1 | 2 | 8.8 |
| Total Organic Carbon (g/100g dry wt) | 0.41 | 9.1 | 17.9 |
| Total Nitrogen (g/100g dry wt) | <0.13 | 0.85 | 1.95 |
| Total Phosphorus (g/kg dry wt) | 2260 | 888 | 2010 |
| Iron (g/kg dry wt) | 51700 | 74100 | 19600 |
| Manganese (g/kg dry wt) | 2750 | 991 | 497 |
| Aluminum (g/kg dry wt) | 19000 | 24600 | 19300 |
| Calcium (g/kg dry wt) | 3990 | 3030 | 4410 |
| Lead (g/kg dry wt) | 32.4 | 33.1 | 14.1 |
| Copper (g/kg dry wt) | 23 | 21.3 | 23.3 |
| Cadmium (g/kg dry wt) | 0.11 | 0.15 | 0.15 |
| Zinc (g/kg dry wt) | 107 | 46.5 | 63.3 |
| Sulfur (g/kg dry wt) | No results | 4950 | 9170 |
| Grain Size ≥ 63 µm (%) |  | 57.74 | 27.93 |
| Grain Size 2 to 63 µm (%) |  | 42.26 | 72.07 |
| Grain Size ≤ 2 µm (%) |  | 0 | 0 |

**Methods for eDNA normalisation to gene copies per litre and gene copies per gram (for Figure S3):**

**Water content determination**

Subsamples of sediment replicates were pooled for each site (0.5 g per site), transferred into pre-weighed glass vials, lyophilized (Gamma 1–16 LSC freeze-dryer; Martin Christ Gefriertrocknungsanlagen, Osterode am Harz, Germany), and re-weighed. The water content was determined using the following formula:

| $Water content of sediment= \frac{wet weight \left( g \right)-dry weight \left( g \right)}{wet weight \left( g \right)}$ | (1) |
| --- | --- |

**Droplet digital PCR normalisation**

Fish gene copy concentrations were then standardized to fish gene copy numbers per gram of dry sediment or per liter of water using the following formulas:

| $Gene copies (sediment) = \frac{ddPCR\times\frac{22.45 \mu L}{6 \mu L}\times100 \mu L}{sed. weight\times(1 - water content)}$ | (2) |
| --- | --- |
| $Gene copies (water) = \frac{ddPCR\times\frac{22.45 \mu L}{6 \mu L}\times100 \mu L}{volume filtered}$ | (3) |

where gene copies = fish gene concentrations (12S rRNA gene for perch and 16S rRNA gene for rudd), ddPCR = concentration of gene copies per µL, 22.45 µL = total reaction volume, 6 µL = volume of DNA template added to the PCR reaction, 100 µL = volume of DNA eluted after extraction, sed. weight = exact weight of each sample extracted for DNA (~3 g), water content = water content of the core subsample in % from Equation (1), and volume filtered = 500 mL.


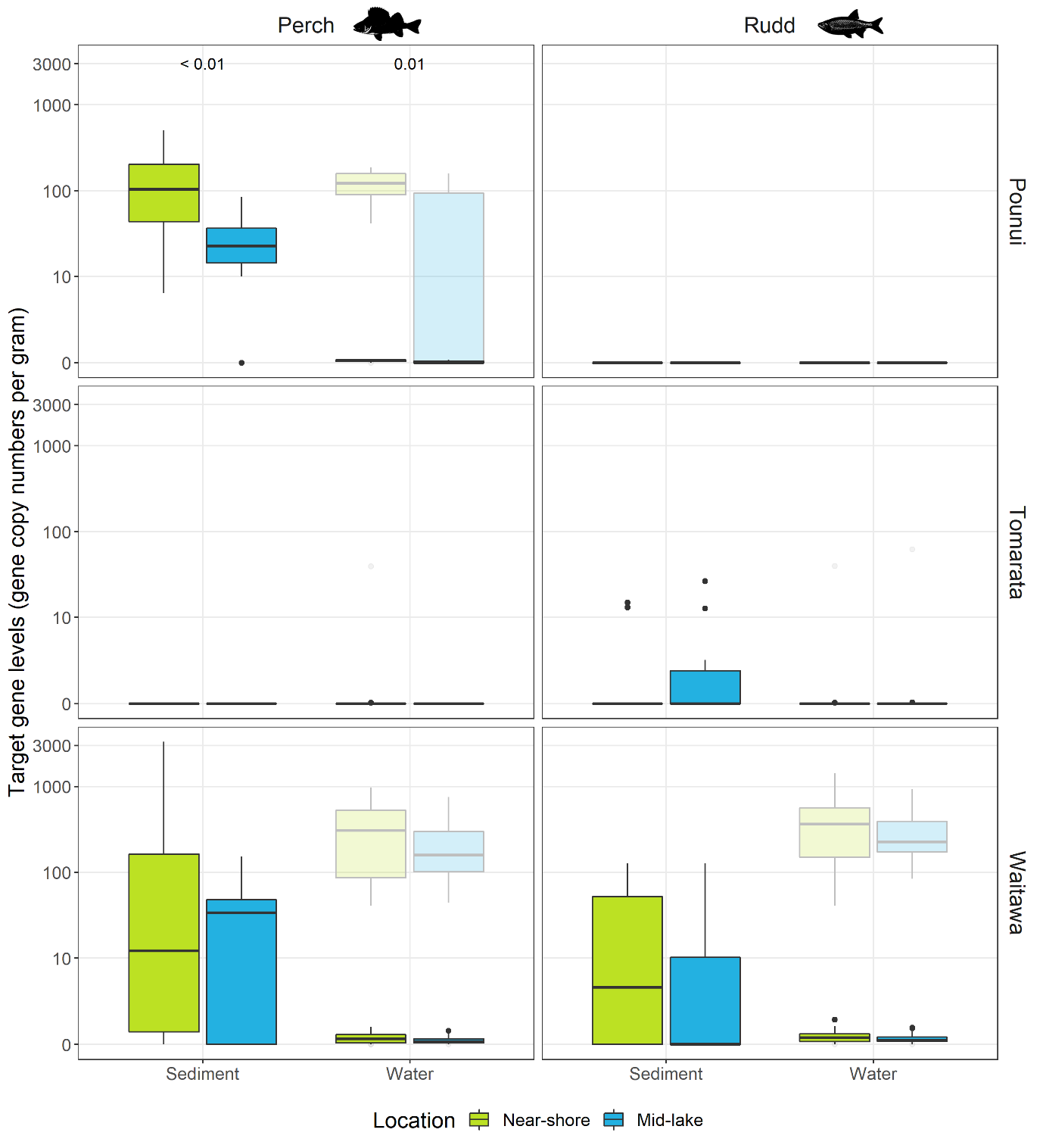


**Figure S3:** Gene copy levels for each fish species per lake (12S rDNA for perch and 16S rDNA for rudd), separated by sampling method (sediment and water) and sampling location (near-shore and mid-lake). Gene levels in water were plotted based on an equivalence of 1 g of water and 1 mL of water. Shaded boxplots indicate eDNA levels in water multiplied by 1,000 (concentrations per L) to visualise difference across locations. Target gene levels are plotted on a log10 scale with null values transformed to one.

**Table S5**: Ranking of the 16 model variants tested for perch eDNA in Lake Pounui made by the PRESENCE software. The best model has the lower Akaike Information Criterion (AIC) and is displayed on the first row (highlighted in blue). Parameters are constant unless indicated otherwise. Abbreviations as follow: psi = Ψ (large-scale occupancy), theta = θ (small-scale occupancy), p = detection probability, m = sampling method (water or sediment), Littoral = location (near-shore or mid-lake). M*Littoral indicates a method and location interaction.


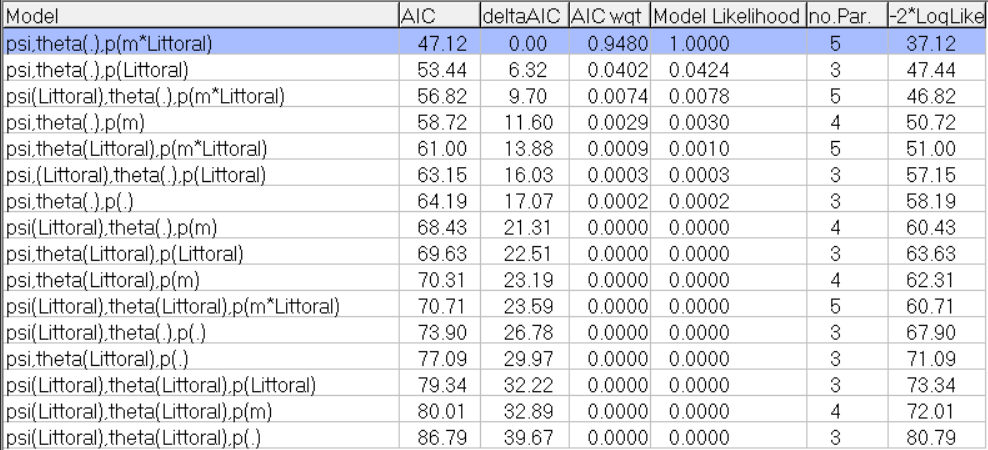


**Table S6**: Ranking of the 16 model variants tested for rudd eDNA in Lake Tomarata made by the PRESENCE software. The best model has the lower Akaike Information Criterion (AIC) and is displayed on the first row (highlighted in blue). Parameters are constant unless indicated otherwise. Abbreviations as follow: psi = Ψ (large-scale occupancy), theta = θ (small-scale occupancy), p = detection probability, m = sampling method (water or sediment), Littoral = location (near-shore or mid-lake). M*Littoral indicates a method and location interaction.


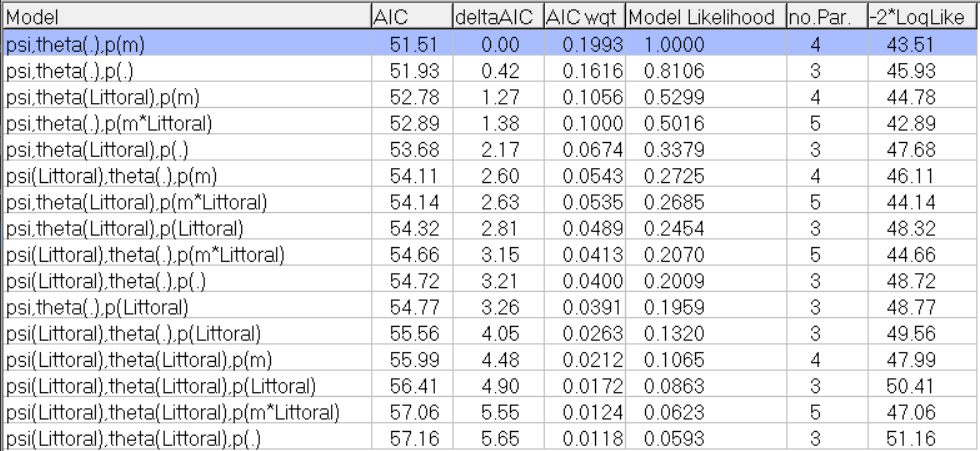


**Table S7**: Ranking of the 16 model variants tested for perch eDNA in Lake Waitawa made by the PRESENCE software. The best model has the lower Akaike Information Criterion (AIC) and is displayed on the first row (highlighted in blue). Parameters are constant unless indicated otherwise. Abbreviations as follow: psi = Ψ (large-scale occupancy), theta = θ (small-scale occupancy), p = detection probability, m = sampling method (water or sediment), Littoral = location (near-shore or mid-lake). M x Littoral indicates a method and location interaction.


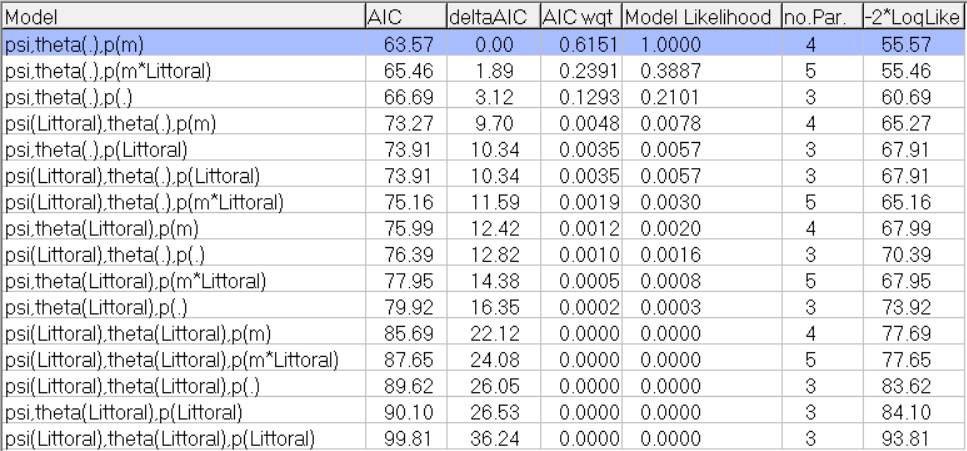


**Table S8:** Ranking of the 16 model variants tested for rudd eDNA in Lake Waitawa made by the PRESENCE software. The best model has the lower Akaike Information Criterion (AIC) and is displayed on the first row (highlighted in blue). Parameters are constant unless indicated otherwise. Abbreviations as follow: psi = Ψ (large-scale occupancy), theta = θ (small-scale occupancy), p = detection probability, m = sampling method (water or sediment), Littoral = location (near-shore or mid-lake). M*Littoral indicates a method and location interaction.


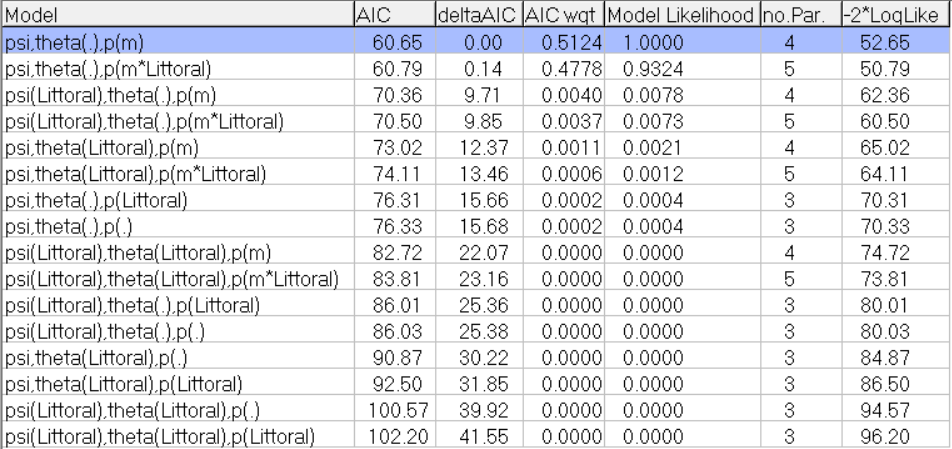


**Table S9**: Simulation for minimum sampling effort for sediment samples – worst-case scenario (p=0.21). The number of sites (S) and replicates (K) were increased until the percentage of empty histories was 0% (no false negative detection) and until the error rates of the estimator were less than 5% (see values in bold).

| Evaluation of design K = 5 S = 6 (TS = 30)  --------------------------------------------------------------------------  estimator performance (excl empty histories)  psi: bias = -0.0968 var = +0.0276 MSE = **+0.0369**  p: bias = +0.0319 var = +0.0084 MSE = **+0.0094**  covar = -0.0072 critA = +0.0464 critD = +2.964e-04  estimator performance (excl also histories leading to boundary estimates)  psi: bias = -0.2228 var = +0.0354 MSE = +0.0850  p: bias = +0.0646 var = +0.0092 MSE = +0.0133  covar = -0.0093 critA = +0.0984 critD = +1.048e-03  empty histories = **0.0%**  boundary estimates = 56.5% |
| --- |

**Table S10**: Simulation for minimum sampling effort for water samples – worst-case scenario (p=0.07). The number of sites (S) and replicates (K) were increased until the percentage of empty histories was 0% (no false negative detection) and until the error rates of the estimator were less than 5% (see values in bold).

| Evaluation of design K = 8 S = 20 (TS = 160)  --------------------------------------------------------------------------  estimator performance (excl empty histories)  psi: bias = -0.1218 var = +0.0335 MSE = **+0.0483**  p: bias = +0.0139 var = +0.0010 MSE = **+0.0011**  covar = -0.0037 critA = +0.0495 critD = +4.207e-05  estimator performance (excl also histories leading to boundary estimates)  psi: bias = -0.2734 var = +0.0338 MSE = +0.1085  p: bias = +0.0297 var = +0.0012 MSE = +0.0020  covar = -0.0039 critA = +0.1106 critD = +2.066e-04  empty histories = **0.0%**  boundary estimates = 55.5% |
| --- |

**Table S11**: Simulation for minimum sampling effort for near-shore sediment samples in Lake Pounui (p=0.99). The simulation was run at p = 0.97 due to the function not being able to handle higher detection probabilities. The number of sites (S) and replicates (K) were increased until the percentage of empty histories was 0% (no false negative detection) and until the error rates of the estimator were less than 5% (see values in bold).

| Evaluation of design K = 2 S = 2 (TS = 4)  --------------------------------------------------------------------------  estimator performance (excl empty histories)  psi: bias = -0.0010 var = +0.0005 MSE = **+0.0005**  p: bias = +0.0022 var = +0.0067 MSE = **+0.0067**  covar = -0.0000 critA = +0.0071 critD = +3.170e-06  estimator performance (excl also histories leading to boundary estimates)  psi: bias = -0.0011 var = +0.0005 MSE = +0.0005  p: bias = +0.0300 var = +0.0000 MSE = +0.0009  covar = +0.0000 critA = +0.0014 critD = +4.790e-07  empty histories = **0.0%**  boundary estimates = 10.8% |
| --- |

**Table S12**: Simulation for minimum sampling effort for perch eDNA using water samples in Lake Waitawa (p=0.89). The number of sites (S) and replicates (K) were increased until the percentage of empty histories was 0% (no false negative detection) and until the error rates of the estimator were less than 5% (see values in bold).

| Evaluation of design K = 2 S = 2 (TS = 4)  --------------------------------------------------------------------------  estimator performance (excl empty histories)  psi: bias = -0.0102 var = +0.0050 MSE = **+0.0051**  p: bias = +0.0113 var = +0.0209 MSE = **+0.0210**  covar = -0.0010 critA = +0.0261 critD = +1.057e-04  estimator performance (excl also histories leading to boundary estimates)  psi: bias = -0.0156 var = +0.0076 MSE = +0.0078  p: bias = +0.1100 var = +0.0000 MSE = +0.0121  covar = +0.0000 critA = +0.0199 critD = +9.465e-05  empty histories = **0.0%**  boundary estimates = 35.1% |
| --- |

**Table S13**: Simulation for minimum sampling effort of rudd eDNA using water samples in Lake Waitawa (p=0.93). The number of sites (S) and replicates (K) were increased until the percentage of empty histories was 0% (no false negative detection) and until the error rates of the estimator were less than 5% (see values in bold).

| Evaluation of design K = 2 S = 2 (TS = 4)  --------------------------------------------------------------------------  estimator performance (excl empty histories)  psi: bias = -0.0037 var = +0.0018 MSE = **+0.0018**  p: bias = +0.0038 var = +0.0150 MSE = **+0.0150**  covar = -0.0002 critA = +0.0168 critD = +2.728e-05  estimator performance (excl also histories leading to boundary estimates)  psi: bias = -0.0048 var = +0.0024 MSE = +0.0024  p: bias = +0.0700 var = +0.0000 MSE = +0.0049  covar = +0.0000 critA = +0.0073 critD = +1.183e-05  empty histories = **0.0%**  boundary estimates = 24.4% |
| --- |

**Table S14:** Comparison of detection of perch DNA using droplet digital PCR (ddPCR) in the sediment samples of Lake Pounui with the PowerSoil kit (0.25g of sediment, columns Binary_0.25 and RawConc_0.25) and with the improved DNA extraction method from Thomson-Laing et al., (2022) (c. 3g of sediment, Binary_3 and RawConc_3). Binary refers to presence/absence of detection and RawConc refers to the raw ddPCR concentration (gene copy numbers per microliter). The replicates where both DNA extraction methods detected perch sedDNA are highlighted in blue.

| Lake | Sample Type | Site | Replicate | Binary_0.25 | RawConc_0.25 | Binary_3 | RawConc_3 |
| --- | --- | --- | --- | --- | --- | --- | --- |
| Pounui | Sediment | 1 | A | 0 | 0 | 1 | 0.17537 |
| Pounui | Sediment | 1 | B | 0 | 0 | 1 | 0.130996 |
| Pounui | Sediment | 2 | A | 0 | 0 | 1 | 0.453754 |
| Pounui | Sediment | 2 | B | 0 | 0 | 1 | 0.110571 |
| Pounui | Sediment | 3 | A | 0 | 0 | 1 | 0.114716 |
| Pounui | Sediment | 3 | B | 0 | 0 | 1 | 0.118007 |
| Pounui | Sediment | 4 | A | 0 | 0 | 0 | 0 |
| Pounui | Sediment | 4 | B | 0 | 0 | 1 | 0.055195 |
| Pounui | Sediment | 5 | A | 0 | 0 | 0 | 0 |
| Pounui | Sediment | 5 | B | 1 | 0.0936 | 1 | 0.17008 |
| Pounui | Sediment | 6 | A | 0 | 0 | 1 | 0.055142 |
| Pounui | Sediment | 6 | B | 0 | 0 | 1 | 0.08136 |
| Pounui | Sediment | 7 | A | 0 | 0 | 1 | 0.052818 |
| Pounui | Sediment | 7 | B | 0 | 0 | 1 | 0.245246 |
| Pounui | Sediment | 8 | A | 0 | 0 | 1 | 0.204612 |
| Pounui | Sediment | 8 | B | 0 | 0 | 1 | 0.208114 |
| Pounui | Sediment | 9 | A | 1 | 0.0795 | 1 | 0.467441 |
| Pounui | Sediment | 9 | B | 0 | 0 | 1 | 1.883944 |
| Pounui | Sediment | 10 | A | 0 | 0 | 1 | 0.262412 |
| Pounui | Sediment | 10 | B | 0 | 0 | 1 | 0.534346 |
| Pounui | Sediment | 11 | A | 0 | 0 | 1 | 0.104214 |
| Pounui | Sediment | 11 | B | 0 | 0 | 1 | 0.154328 |
| Pounui | Sediment | 12 | A | 0 | 0 | 1 | 0.154791 |
| Pounui | Sediment | 12 | B | 0 | 0 | 1 | 0.10108 |
| Pounui | Sediment | 13 | A | 1 | 0.0796 | 1 | 0.673854 |
| Pounui | Sediment | 13 | B | 1 | 0.0814 | 1 | 1.198951 |
| Pounui | Sediment | 14 | A | 0 | 0 | 1 | 0.055186 |
| Pounui | Sediment | 14 | B | 0 | 0 | 1 | 0.192223 |
